# Conformationally restricted calpain inhibitors†
†Electronic supplementary information (ESI) available. See DOI: 10.1039/c5sc01158b



**DOI:** 10.1039/c5sc01158b

**Published:** 2015-08-24

**Authors:** S. E. Adams, E. J. Robinson, D. J. Miller, P. J. Rizkallah, M. B. Hallett, R. K. Allemann

**Affiliations:** a School of Chemistry , Cardiff University , Main Building, Park Place , Cardiff , UK CF10 3AT . Email: allemannrk@cardiff.ac.uk ; Fax: +44 (0) 29 208 74030 ; Tel: +44 (0) 29 2087 9014; b Institute of Infection & Immunology , School of Medicine , Heath Campus , Cardiff , UK CF14 4XN

## Abstract

The cysteine protease calpain-I is linked to several diseases and is therefore a valuable target for inhibition. Selective inhibition of calpain-I has proved difficult as most compounds target the active site and inhibit a broad spectrum of cysteine proteases as well as other calpain isoforms. Selective inhibitors might not only be potential drugs but should act as tools to explore the physiological and pathophysiological roles of calpain-I. α-Mercaptoacrylic acid based calpain inhibitors are potent, cell permeable and selective inhibitors of calpain-I and calpain-II. These inhibitors target the calcium binding domain PEF(S) of calpain-I and -II. Here X-ray diffraction analysis of co-crystals of PEF(S) revealed that the disulfide form of an α-mercaptoacrylic acid bound within a hydrophobic groove that is also targeted by a calpastatin inhibitory region and made a greater number of favourable interactions with the protein than the reduced sulfhydryl form. Measurement of the inhibitory potency of the α-mercaptoacrylic acids and X-ray crystallography revealed that the IC_50_ values decreased significantly on oxidation as a consequence of the stereo-electronic properties of disulfide bonds that restrict rotation around the S–S bond. Consequently, thioether analogues inhibited calpain-I with potencies similar to those of the free sulfhydryl forms of α-mercaptoacrylic acids.

## Abstract 2

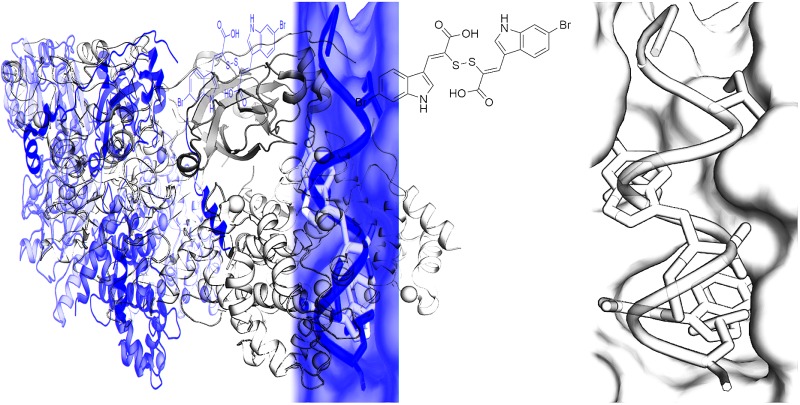
Oxidised α-mercaptoacrylic acid derivatives are potent conformationally restricted calpain-I inhibitors that mimic the endogenous inhibitor calpastatin.

## Introduction

Calpain-I and calpain-II are the two most studied members of a family of calcium dependent cysteine proteases that currently comprises fifteen identified gene products in humans.^1–3^ These heterodimeric proteases are composed of a large subunit with a molecular mass of ∼80 000 and a small subunit of mass ∼30 000. Calpain-I and calpain-II share a small subunit, which consists of two domains, a penta-EF hand calcium binding domain PEF(S) and a glycine rich domain that is thought to interact with cellular membranes;^4^ the large subunits of calpain-I and -II possess 62% sequence similarity in humans^5^ and comprise four distinct domains, a N-terminal anchor helix, the active site domain (CysPc), a domain that resembles the C2 membrane binding domains of phosphokinases and is hence known as the C2L domain,^2,6^ and a second penta-EF hand calcium binding domain known as PEF(L). PEF(L) is the domain that determines the concentration of calcium required for protease activation, which is the discriminating factor between the two isoforms.^1,7^ Calpain-I is activated *in vitro* by approximately 50 μM Ca^2+^, whereas calpain-II requires approximately 350 μM Ca^2+^ for activation.^1^

Numerous physiological processes have been linked with calpain-I and -II, including cell motility,^8–10^ apoptosis^11,12^ and progression through the cell cycle^13^ but the precise roles of these proteases remain poorly understood, which is at least in part due to a lack of specific inhibitors that allow selective knockout or knock-down of their cellular activities.^14^ Other techniques such as microinjection of a surplus of the enzymes or the release of calcium ions into cells have been used to activate the enzymes to explore their cryptic roles *in vivo*. The majority of synthetic inhibitors available to examine the role of these isoforms are generic cysteine protease inhibitors that react with the active site cysteine and hence show little discrimination between calpain isoforms or indeed other cysteine proteases such as caspases or cathepsins.^15–17^ The calpain system includes an endogenous inhibitor, calpastatin (CAST), a large protein that only binds to members of the calpain family that form a heterodimeric complex, hence discriminating between calpains and other cysteine proteases. Specific cell permeable inhibitors of calpain(s) that target the CAST binding sites could be used as valuable cell-biological tools for the elucidation of the cryptic physiological and pathophysiological roles of calpain isoforms and potentially as drugs to treat conditions such as autoimmune diseases, ischemic stroke damage and cancer.^14,18,19^

**Fig. 1:**
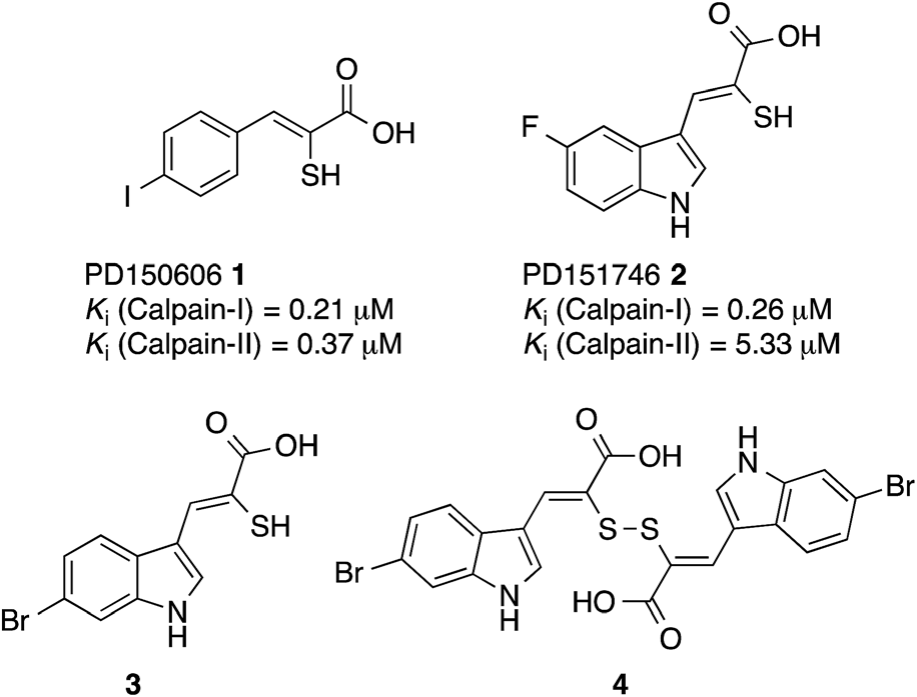
Structures of PD150606 (**1**), PD151746 (**2**), (*Z*)-3-(6-bromoindol-2-yl)-2-mercaptoacrylic acid (**3**) and (2*Z*,2′*Z*)-2,2′-disulfanediylbis(3-(6-bromoindol-3-yl)acrylic acid) (**4**).^20,24^

A previous investigation led to the discovery of novel, isoform-selective inhibitors that target calpain through interactions with allosteric binding sites.^17^ The α-mercaptoacrylic acids PD150606 (**1**) and PD151746 (**2**) (Fig. 1)^20^ bind to the calcium binding domain of calpain rather than the active site and show modest selectivity for calpain-I over calpain-II.^21,22^ The hydrophobic pocket of PEF(S) targeted by these inhibitor is also bound by the inhibitory region C of calpastatin domain IV, where Leu 660 is embedded in the pocket.^23^ Based on compounds **1** and **2**, potent cell permeable inhibitors of calpain-I that inhibit the cell spreading action of live neutrophils *in vitro* were synthesised.^24,25^ A single co-crystal X-ray structure showed that like PD150606 the new compounds bound to the calcium binding domain PEF(S).^24,26^ The thiol and carboxylic acid groups of the inhibitors are critical for activity^20,27^ but somewhat surprisingly these functional groups point away from the protein into solution. Here we report that oxidation of the sulfhydryls of these α-mercaptoacrylic acids to form disulfides leads to inhibitors with greatly enhanced potency, where one half of the compound targets the hydrophobic CAST binding groove of PEF(S). This previously unexplored mode of action opens the way for the development of a new generation of stable and selective inhibitors of calpain-I.

## Results and discussion

**Fig. 2:**
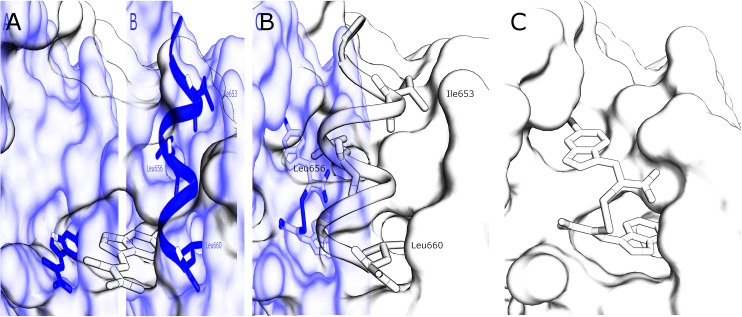
(A) Surface representation of chain A of PEF(S) bound to **3** in the hydrophobic pocket that binds Leu 660 (PDB ; 4WQ2). (B) Inhibitory region C of the CAST domain IV bound to PEF(S); Ile 653, Leu 656 and Leu 660 are highlighted (PDB ; 3BOW).^23^ (C) **4** bound to chain A of PEF(S); a single binding mode of **4** is represented (PDB ; 4WQ3).

The interaction of (*Z*)-3-(6-bromoindole-3-yl)-2-mercaptoacrylic acid (**3**) (Fig. 1) with human PEF(S) was investigated by single crystal X-ray crystallography. Human PEF(S) was produced in *E. coli*, purified to apparent homogeneity, crystallised and soaked with **3** as previously described for other α-mercaptoacrylic acids.^26,28^ Similar to other PEF(S) α-mercaptoacrylic acid co-crystal structures, **3** bound to the hydrophobic pocket of PEF(S) in the region that also binds Leu 660 of the CAST domain IV inhibitory region C. This is situated between the second and fourth α-helices (Fig. 2).^21–23,26^ The hydrophobic and carbonyl portions of **3** match the spatial orientation of Leu 660, but many unexplored possibilities appear to exist for hydrophobic contacts corresponding to preceding residues in the α-helix of CAST inhibitory region C (Fig. 2).^23^ The hydrophobic pockets that would otherwise hold the side chains of Leu 656 and Ile 653 of the inhibitory helix are nearby and should be available to bind additional hydrophobic groups of a designed inhibitor (Fig. 2). The close proximity of these lipophilic amino acids to one another suggested that disulfide versions of α-mercaptoacrylic acids might be able to mimic the binding mode of calpastatin. Close inspection of the structure of the inhibitory region C of the CAST domain IV bound to PEF(S) and molecular docking experiments revealed the possibility that due to the stereoelectronic constraints of sulfur–sulfur bonds the two aromatic rings of a disulfide linked linked form of **3** might fit into the two adjacent hydrophobic pockets of PEF(S); in particular the aromatic groups of such a disulfide based compound may interact with the pockets that bind Leu 656 and Leu 660 of calpastatin. To test this proposal, the oxidised form of **3**, (2*Z*,2′*Z*)-2,2′-disulfanediylbis(3-(6-bromoindol-3-yl)acrylic acid), (**4**, Fig. 1), was generated and the single crystal X-ray structure of **4** bound to PEF(S) solved.

The rate of conversion of **3** to its disulfide form was dependent upon both the concentration of the compound and the nature of the solution. UV-VIS spectroscopy showed that **3** (100 μM in 100 mM K_i_PO_4_, pH 7.0) was completely converted to its oxidised form **4** in 3 hours at room temperature and was easily reduced to the sulfhydryl form by addition of tris(2-carboxyethyl)phosphine (TCEP) (see ESI†). For crystallography, **3** (50 mM) was dissolved in DMSO and left at room temperature for 24 hours, after which time oxidation was complete according to ^1^H-NMR spectroscopy (see ESI†). Compound **4** was then added to preformed crystals of PEF(S).

The electron density map revealed that the ligand adopts two different conformations in each subunit of the PEF(S) homodimer, therefore a total of four conformations of the ligand were observed in the asymmetric unit of the crystal structure. The unique geometry of the disulfide bond is observed in all of the conformations ensuring that both pockets of the hydrophobic groove of PEF(S) are explored. One of the 6-bromoindole rings of **4** is bound in the hydrophobic binding pocket that also binds **3**. The aromatic ring is capable of moving within the pocket, adopting two different conformations with the ring flipped by approximately 180° relative to the other as observed in chain A of PEF(S) (Fig. 2). The second indole ring of **4** interacts with a hydrophobic groove on the surface of PEF(S), where the α-helix of CAST inhibitory region C binds with PEF(S).^21^ Two different binding orientations of the ligand are observed, allowing for different protein residues to interact with the ligand. In chain A, the second 6-bromoindole ring resides in a hydrophobic pocket where Leu 656 of CAST also binds (Fig. 2), whereas in chain B it targets the hydrophobic groove that binds the peptide backbone of CAST inhibitory region C (Fig. 2).^23^

**Fig. 3:**
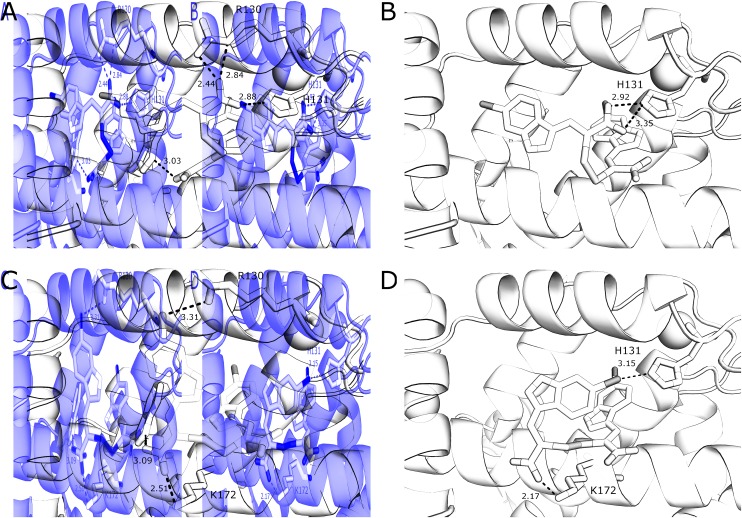
Comparison of the two conformations **4** (cyan) bound to chain A (yellow) of the PEF(S) homodimer, (A) and (B) respectively, and chain B (green) of the homodimer, (C) and (D) respectively. Residues that form halogen bonds, hydrogen bonds and electrostatic interactions are highlighted and important bonds are indicated by dashed lines, distances are shown in Å (PDB ; 4WQ2 and ; 4WQ3).

With the exception of a hydrogen bond between the carboxylate of **3** and the side chain amide group of Gln175 in chain B, van der Waals forces are the main interactions observed between **3** and the residues of both monomers of the protein. A greater number of interactions form between **4** and residues of PEF(S) than with **3** and the protein, including a number of electrostatic interactions and hydrogen bonds. In chain A, six hydrophilic interactions between the two conformations of **4** and Arg130, His131 and Trp168 are observed (Fig. 3). An electrostatic bond forms between the carboxylate group of **4** and the positively charged guanidinium group of Arg130. Hydrogen bonds are observed between the carboxylates of the ligand and the side-chain NH groups of His131 and Trp168 (Fig. 3). A different electrostatic interaction is seen between **4** bound to chain B between a carboxylate group of the ligand and the NH_3_^+^ group of Lys172 with distances of 2.17 Å and 2.51 Å for the two conformations (Fig. 3). Interactions form between the bromine atoms of **4** and hydrophilic groups in chain B; one bromide interacts with Arg130 and the second with His131 with Br–N distances of 3.31 Å and 3.15 Å, respectively (Fig. 3). The greater number of interactions observed between compound **4** and PEF(S) suggests tighter binding to PEF(S). The structure of **4** bound to PEF(S) prompted a re-evaluation of the inhibitory potency of the α-mercaptoacrylic acids previously synthesised.^24^ Previously, no special measures had been taken to ensure that the compounds were in oxidised or reduced form during the assays. The oxidised and reduced compounds were now assayed against calpain-I to determine their affinity for PEF(S).

**Fig. 4:**
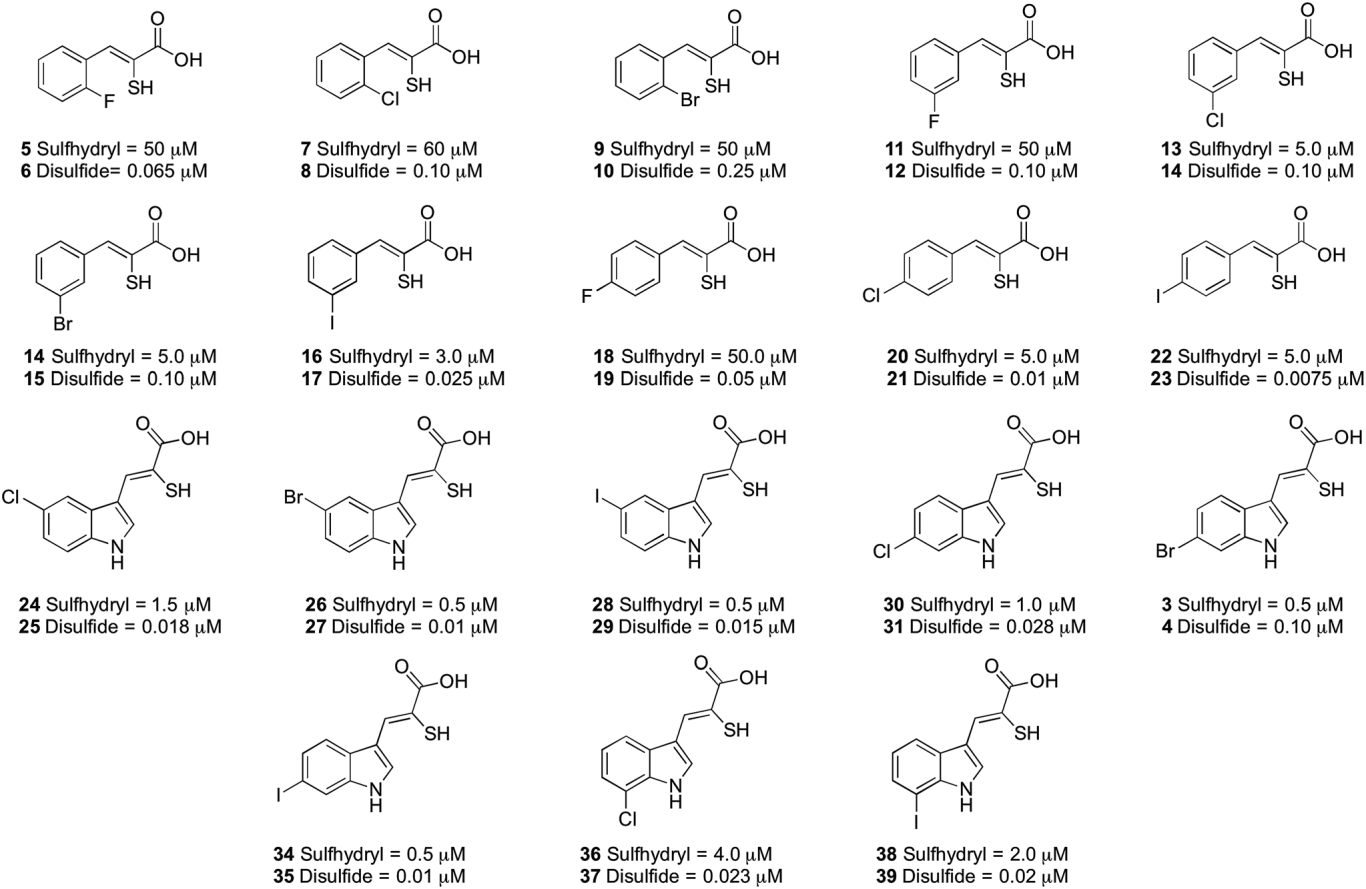
IC_50_ values (μM) for the α-mercaptoacrylic acid compounds tested in FRET based inhibition assays in the presence (sulfhydryl) and absence (disulfide) of 10 mM DTT.

The inhibitory properties of a series of oxidised and reduced α-mercaptoacrylic acid derivatives towards calpain-I (Fig. 4) were assessed in a FRET-based assay in the presence and absence of 10 mM dithiothreitol (DTT).^24,25^ To ensure complete oxidation, compounds were left in the assay solution for 3 hours prior to performing the assay (*vide supra* and ESI†). In the absence of inhibitors there was no observable difference in enzyme activity when assayed under both oxidising and reducing conditions.

When DTT was present, IC_50_ values were in the micromolar range for both phenyl and indole based α-mercaptoacrylic acid derivatives (Fig. 4). As observed previously,^24^ the position of the halogen in the aromatic ring for phenyl based inhibitors was important for the inhibitory potency. All 2-substituted compounds have IC_50_ values of ∼50 μM whereas compounds that are substituted in the 3- or 4-position are about one order of magnitude more potent. Also, the type of halogen is important for the potency for the inhibitor in that F substituted compounds are approximately 10-fold weaker inhibitors than the corresponding Br and I substituted inhibitors. This trend does not hold for Cl and Br substituents in the 2 position, which bind with affinities of ∼60 μM and ∼50 μM, respectively (Fig. 4). The indole based α-mercaptoacrylic acid derivatives showed IC_50_ values in the low micromolar range in the presence of DTT and there was no obvious trend with respect to position or type of halogen (Fig. 4). The IC_50_ values reported here are higher than previously reported for the same compounds, most likely due to the presence of poorly defined mixtures of reduced and oxidised mercaptoacrylic acids in previous experiments.^14–17,20,24^

For the oxidised compounds IC_50_ values were in the nanomolar range, a 10- to 200-fold increase in potency, indicating that the thiol group is not important for inhibition of calpain-I. The IC_50_ values for the phenyl derivatives range from 7.5 to 250 nM. The position of the halogen was important for the potency of the inhibitor with a trend similar to that observed for the reduced compounds. Iodo substituted compounds (**17** and **23**) showed IC_50_ values of 25 nM and 7.5 nM, respectively (Fig. 4). With the exception of the iodo-substituent the type of halogen appears to have little importance for IC_50_ values. The indole based disulfide compounds show a 10- to 150-fold increase in potency relative to the free sulfhydryls. IC_50_ values ranged from 10 to 28 nM, with the exception of **3**, which was less potent (IC_50_ = 100 nM) (Fig. 4). No discernible trend was observed with respect to the position or type of halogen relative to the potency of the inhibitor (Fig. 4). The most potent inhibitor in this series was the disulfide of PD150606 (**23**), which when tested against calpain-I gave an IC_50_ value of 7.5 nM, much more potent than the *K*_i_ value of 0.21 μM, possibly due to the inhibition of calpain-I being measured in a poorly defined redox buffer (Fig. 4).^20^ The increased potency of PD150606 in the disulfide form **23** led us to an examination of the co-crystal structure PEF(S) with this inhibitor in oxidised form.^21,22^ The structure obtained shows a lower electron density around the model of the ligand **23** to that observed for **4**, though upon comparison of the two structures both ligands adopt similar conformations and form analogous interactions with the protein (see ESI†).

**Fig. 5:**
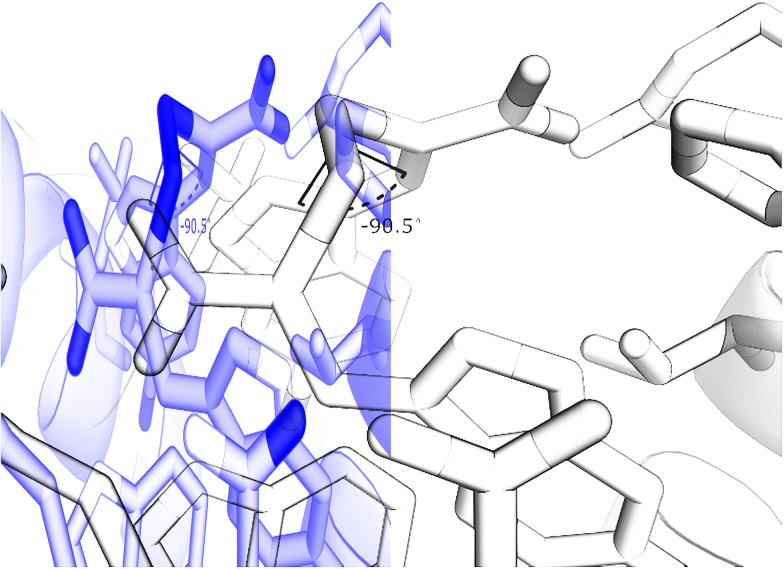
Representation of **4** with a dihedral angle of –90.5° bound to chain A of PEF(S).

The disulfide bond is the only group in the structure of the oxidised α-mercaptoacrylic acids that allows for flexibility within the molecule. However in agreement with the design, this flexibility is limited due to the specific stereoelectronic properties of S–S bonds. Rotation around the disulfide bond is restricted due to the repulsive interactions of the lone pairs of each sulfur atom.^29^ Dihedral angles of aromatic disulfides are typically between 100 and 106°,^29,30^ although the dihedral angles can be both greater and smaller depending upon the substituents.^31^ The dihedral angles of the observed conformations of **4** bound to PEF(S) are 30, –91, 128 and 169. The dihedral angle of –91 in chain A is close to the optimum dihedral angle and in this conformation **4** makes strong interactions with both hydrophobic pockets of PEF(S) (Fig. 5). Disulfide bonds are on average 2.08 Å, approximately 0.5 Å longer than a carbon–carbon single bond. The S–S bond length of **4** bound to PEF(S) ranges from 2.06 to 2.11 Å.^32^ The slightly increased bond length allows for the second aryl group to interact with the hydrophobic pocket that binds Leu 656 of calpastatin. To establish whether the properties of the disulfide bond were indeed responsible for the strong inhibitory of compounds such as **4**, one of the sulfur atoms was replaced with a methylene group to generate a series of thioethers. The relatively unhindered rotation around carbon–sulphur bonds should lead to a greater loss of entropy when they bind to PEF(S) and hence to reduced stability compared to the disulfide complexes.

**Fig. 6:**
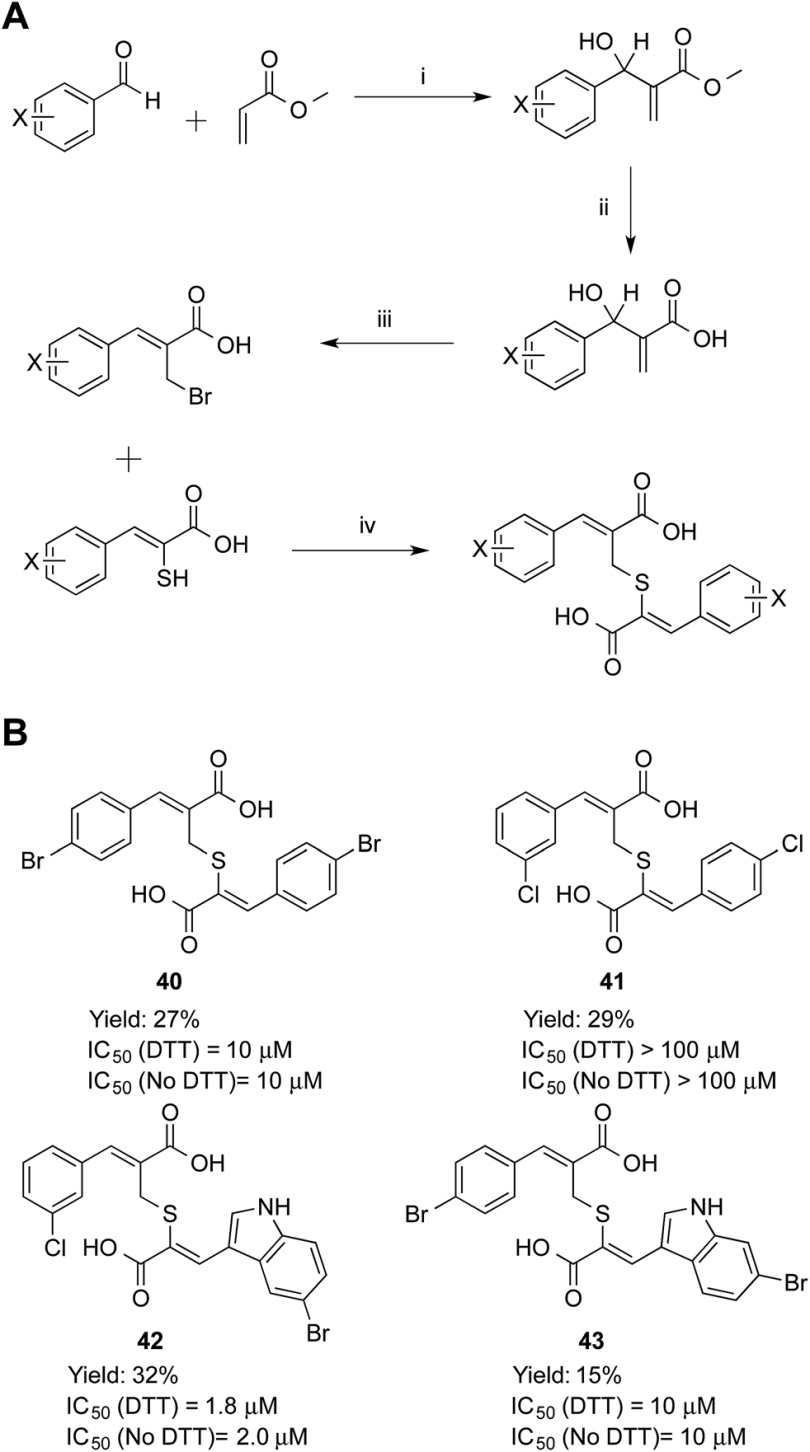
(A) Synthetic route to the thioether calpain inhibitors. (i) DABCO, MeOH, 25 °C, 72 h, (ii) NaOH, MeOH, 25 °C, 16 h, (iii) HBr, H_2_SO_4_, 25 °C, 16 h, (iv) NEt_3_, MeCN, 25 °C, 16 h. (B) Symmetric and asymmetric compounds **40–43**. (*Z*)-3-(4-Bromophenyl)-2-((((*Z*)-2-(4-bromophenyl)-1-carboxyvinyl)thio)methyl)acrylic acid (**40**), (*Z*)-3-(3-chlorophenyl)-2-((((*Z*)-2-(4-chlorophenyl)-1-carboxyvinyl)thio)methyl)acrylic acid (**41**), (*Z*)-3-(5-bromoindol-3-yl)-2-(((*Z*)-2-carboxy-3-(3-chlorophenyl)allyl)thio)acrylic acid (**42**) and (*Z*)-3-(6-bromoindol-3-yl)-2-(((*Z*)-3-(4-bromophenyl)-2-carboxyallyl)thio)acrylic acid (**43**).

Symmetric and asymmetric thioether compounds were synthesised in a four-step procedure (Fig. 6). A halogenated benzaldehyde was reacted with methyl acrylate, followed by base catalysed hydrolysis of the ester and treatment with aqueous hydrogen bromide to generate an allylic bromide derivative, which was used to alkylate an α-mercaptoacrylic acid.^24^ All IC_50_ values of were in the micro-molar range between 1.8 and 10 μM except for **41** (IC_50_ = 100 μM) and DTT had no effect on the potency (Fig. 6). With an IC_50_ value of 1.8 μM the asymmetric **42** was the most potent thioether examined. The IC_50_ values measured were higher than those obtained for all the disulfides examined here and indeed generally higher than the free sulfhydryls. Also, there was no discrimination between the symmetric (**40**) and asymmetric (**41**, **42** and **43**) thioether derivatives with regards to the efficacy of these compounds towards calpain-I.

Inhibition data for the thioether-based compounds supports the importance of the stereoelectronic properties of the S–S bond for the potency of the mercaptoacrylic acid based inhibitors. The greater degree of flexibility of the thioether bond leads to higher entropic penalty when these compounds ‘lock’ into position on PEF(S), which in turn produces higher IC_50_ values. In addition, the decreased bond length of the C–S bond relative to the S–S bond may lead to a suboptimal interaction of the second aromatic ring with the hydrophobic pocket of PEF(S) that binds Leu 656 of CAST. Crystallographic results support this rationalisation of the low PEF(S) binding affinity of the thioethers. Single crystals of PEF(S) were individually soaked with **40**, **42** and **43** and the resulting crystals analysed. The X-ray diffraction data indicated only partial occupancy of the calpastatin binding site. Models representing compounds **40**, **42** and **43** were placed into each of the 2*F*_0_ – *F*_c_ maps revealing a maximum occupancy of the ligands within the electron density of 30% (see ESI†).

## Conclusions

The work described here reveals a novel mechanism for inhibition of calpain-I by α-mercaptoacrylic acid derivatives. Based on the X-ray crystal structure of PEF(S) bound to (*Z*)-3-(6-bromoindol-2-yl)-2-mercaptoacrylic acid (**3**), oxidised α-mercaptoacrylic acids such as **4** were designed to target the full calpastatin binding cavity. The restricted geometry of the disulphide bond facilitated a larger number of favourable binding interactions with PEF(S) so that the two aromatic rings of **4** interact with the hydrophobic pockets that bind Leu 660 and Leu 656 of calpastatin. All disulfide linked compounds acted as potent inhibitors of calpain-I and were up to 200 times more potent than their reduced counterparts. The dramatic increase in potency of the oxidised inhibitors can be tentatively explained by the stereoelectronic properties of S–S bonds such as the reduced rotations around the S–S bond and the approximately 90° dihedral angles. Accordingly, thioether analogues were much less potent than their disulfide counterparts.

These results open the way to the development of inhibitors that combine the conformational restrictions of the S–S bond with reduced sensitivity to reduction. Diselenides are characterised by dihedral angles of 101–106° and bond lengths of approximately 2.3 Å, values similar to those found in disulfides. However, diselenides are more stable towards reduction than disulfides.^33–35^ The incorporation of diselenide bonds could therefore lead to highly potent drugs and cell biology tools that are stable to the reducing conditions found in the cellular environment.
