# Supplementary material for: Conformationally restricted calpain inhibitors
Source: Chem Sci. 2015 Aug 24;6(12):6865–71. doi: 10.1039/c5sc01158b (PMC5508670; doi:10.1039/c5sc01158b)
Supplement: Supplementary file 1 [file SC-006-C5SC01158B-s001.pdf]

Electronic Supplementary Material (ESI) for Chemical Science.
This journal is © The Royal Society of Chemistry 2015

Electronic Supplementary Information

Conformationally Restricted Calpain Inhibitors

S. E. Adams[a], E. J. Robinson[b], D. J. Miller[a], P. J. Rizkallah[b], M. B. Hallett[b] and R. K. Allemann[a]

a) School of Chemistry, Main Building, Park Place, Cardiff University, Cardiff, UK, CF10 3AT. b) Welsh Heart Research Institute, School of Medicine, Heath Hospital, Cardiff University, Cardiff, UK, CF14 4XN.

Table of Contents

1. Protein Expression, Crystallisation and FRET Based Inhibition Assay Methods……………………….1
2. Crystallography Data Statistics……………………………………………………………………..2
3. Inhibitor-Protein Interaction Analysis……………………………………………………………2-3
4. Protein R.M.S.D calculations……………………………………………………………………..4-8
5. Compound Oxidation Analysis…………………………………………………………………….8-12
6. Organic Synthesis……………………………………………………………………………...13-23
7. Inhibition of calpain-I with (2Z,2'Z)-2,2'-disulfanediylbis(3-(6-bromoindol-3-yl)acrylic acid)…24
8. Thioether X-ray Crystallography Data…………………………………………………………25-26
9. Oxidised PD150606 crystal complex with PEF(S)……………………………………………27-28
10. References……………………………………………………………………………………28-29

Protein Expression, Crystallisation and FRET Based Inhibition Assay Methods

Expression, purification and crystallisation of PEF(S)

The codon optimised gene encoding human PEF(S) was purchased from Epoch Biolabs (Texas, USA) in a pET21d vector. Human PEF(S) was produced in *E. coli* BL21-CodonPlus(DE3)-RP (Agilent Technologies) and purified and crystallized using the same procedure previously described the for PEF(S) from *Sus scrofa*.[1] The crystals took approximately one week to grow. Prior to data collection glycerol was added to the drop containing the crystals to a total concentration of 20% (w/v). The crystals were then harvested and flash frozen in liquid nitrogen.

Soaking the inhibitors into the preformed crystals of PEF(S) was carried out 24 h prior to the harvesting of crystals. Solutions of (*Z*)-3-(6-bromoindol-3-yl)-2-mercaptoacrylic acid (16 mM) in the precipitant solution (50 mM sodium cacodylate, 12.5% PEG6000, 20 mM calcium chloride and 10 mM DTT at pH 7.0) were prepared immediately before soaking. 1 µl of the 16 mM solutions were added to the drops containing the crystals to a total concentration of 2 mM in the drop.

Data Collection and Phasing

The diffraction data was collected at Diamond Light Source (Oxford, UK, beamlines I03 and I04-1) at a temperature of 100 K. The wavelengths used for diffraction were 0.976 Å (PEF(S)-**3**)and 0.920 Å ((PEF(S)-**4**) with a Pilatus pixelated detector. The raw diffraction images were processed through the xia2 data-reduction system.[2] The data were scaled, reduced and analysed using Scala[3] and Aimless[4] from the CCP4i package (Collaborative Computational Project number 4).[5]

Molecular Replacement and Refinement

The structures were solved with molecular replacement using Phaser (CCP4i).[6] The search model was derived from the structure of PEF(S) (PDB:4PHJ).[6, 7] The solution obtained was adjusted with the COOT program (Crystallographic Object-Oriented Toolkit)[8] for molecular model building and completion, and the model was refined further with the Refmac5 refinement program.[9] The models for the small molecules **3** and **4**, were created with ProDrg.[10]

FRET based Inhibition Assay

This assay employed a peptide from the calpain-1 substrate α-spectrin, with fluorescein at one terminus internally quenched by DABCYL and the other (H_2N-K(FAM)-EVYGMMK(DABCYL)-OH).[11] Cleavage by calpain-1 occurs between the Tyr-Gly residues and results in enhanced fluorescence as the quenching effect is relieved. The assays were performed using purified porcine Calpain-1 (CalBiochem, 10 nM) and fluorogenic calpain-1 substrate (Merck, 1 µM) in an assay buffer consisting of HEPES (10 mM) pH 6.8; EDTA (0.5 mM); bovine serum albumin (0.1%). The assay was performed using a fluorescent plate reader (BMG Optistar) in an assay volume of 100 µl at a temperature of 37°C, using an excitation band pass filter centred at 485 nm and emission detected at 520 nm. The compounds to be tested for inhibition were added to the assay mixture before the reaction was initated by the addition of $CaCl_2$ (5 mM). None of the compounds had significant fluorescence at this wavelength and correction for this was unnecessary. The compounds were dissolved in DMSO at 50 mM and diluted into assay buffer to give range of concentrations from 5 nM to 50 µM. In each assay run, the effect of DMSO alone over the concentration used was also measured. Although there was no effect of DMSO at lower concentrations, in some assay runs, DMSO at 0.005%-0.5% produced some inhibitory effect. This DMSO effect (which was only relevant for compounds with poor inhibitory ability) was subtracted before constructing the inhibition curves.

FRET based inhibition assay (DTT addition)

The same procedure was used as described above except with assay buffer containing HEPES (10 mM) pH 6.8; EDTA (0.5 mM); bovine serum albumin (0.1%) and DTT (10 mM).

Crystallography Data Statistics

The data statistics for the structures of PEF(S) - **3** and PEF(S) - **4**.

	PEF(S)-3	PEF(S)-4
**Data Collection**		
X-ray source	DLS I03	DLS I04-1
Space Group	$P12_11$	$P12_11$
Cell Dimensions		
a, b, c (Å)	49.72, 78.43, 56.30	49.56, 79.31, 57.02
α, β, γ (°)	90.00, 91.12, 90.00	90.00, 91.47, 90.00
Wilson B-factor (A^2)	24.1	23.8
Resolution (Å)	41.99-1.64 (1.68-1.64)	39.66-1.79 (1.84-1.79)
Unique Reflections	51550 (3749)	40565 (3101)
Multiplicity	3.7 (3.6)	3.7 (3.9)
Completeness (%)	97.5 (96.5)	97.6 (99.8)
Mean $I/\sigma I$	16.1 (1.7)	11.0 (2.0)
R_{merge}	0.052 (0.901)	0.071 (0.634)
**Refinement**		
Resolution/Å	41.99-1.64	39.65-1.79
No. Reflections	48906	38513
R_{work} / R_{free}	0.170/0.197	0.178/0.215
No. atoms		
Protein	2993	2896
Ligand/ion	56	136
Water	157	297
B-factors/Å^2		
Protein	33.6	28.9
Ligands	55.7	45.7
Ions	32.3	25.9
Water	41.5	34.7
r.m.s.deviations		
Bond length/Å	0.019	0.021
Bond angles/°	1.967	2.039
PDB code	4WQ2	4WQ3

Interactions under 3.5 Å between the protein and the inhibitors

(*Z*)-3-(6-Bromoindol-3-yl)-2-mercaptoacrylic acid (3)

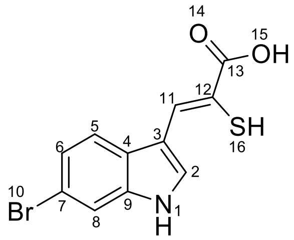

Chain A			Chain B		
Inhibitor Atom	Residue/Atom	Distance/Å	Inhibitor Atom	Residue/Atom	Distance/Å
10	V128/Cγ1	3.20	10	V128/Cγ1	3.33
12	Q175/Nε2	3.38	12	Q175/Cδ	3.31
12	Q175/Cδ	3.19	12	Q175/Oε1	3.23
12	Q175/Oε1	3.26	14	Q175/Oε1	3.21
14	H131/Cε1	3.17	14	Q175/Nε2	3.48 (hydrogen bond)

(2Z,2’Z)-2,2’-disulfanediylbis(3-(6-bromoindol-3-yl)acrylic acid) (4)

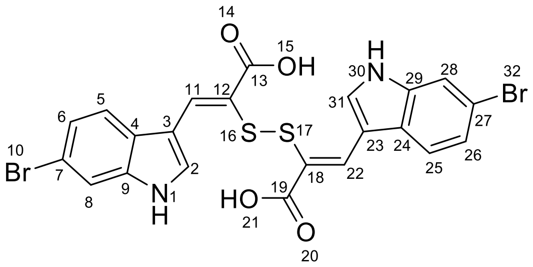

Chain A			Chain B		
Inhibitor Atom	Residue/Atom	Distance/Å	Inhibitor Atom	Residue/Atom	Distance/Å
1B*	Q175/Oε1	3.39	5A	H131/Nε2	3.47
1B	H131/Cγ	3.41	6A	V127/Cγ1	3.46
1B	H131/Cε1	3.47	6B	V127/Cγ1	3.43
1B	H131/Nε2	3.34	7B	V127/Cγ1	3.41
1B	H131/Cδ2	3.32	10A	V127/Cγ1	2.78
2B	Q175/Cδ	3.29	10B	V127/Cγ1	2.85
2B	Q175/Oε1	3.09	11A	Q175/Oε1	3.48
2B	H131/Cε1	3.28	11A	Q175/Cδ	3.38
2B	H131/Nε2	3.41	11A	Q175/Nε2	3.44
7A	V127/Cγ1	3.48	14A	H131/Cε1	3.21
8A	V127/Cγ1	3.47	16B	K172/Cδ	3.32
14A	K172/Cδ	3.19	16B	K172/Cε	3.35
14A	W168/Nε1	3.03 (hydrogen bond)	19B	Q100/Oε1	3.48
14B	H131/Cε1	3.22	19B	K172/Nζ	3.30
16B	K172/Nζ	3.34	20A	K172/Cδ	3.26
19B	H131/Nε2	3.44	20A	K172/Cε	2.96
20A	R130/Nε	2.84 (salt bridge)	20A	K172/Nζ	2.51 (salt bridge)
20A	R130/Cζ	3.03	20B	K172/Cε	3.19
20A	R130/Nη2	2.44 (salt bridge)	20B	K172/Nζ	2.17 (salt bridge)
20B	H131/Nε2	2.88 (hydrogen bond)	21A	Q100/Oε1	3.38
21A	H131/Cε1	3.41	21A	W168/Nε1	3.09 (hydrogen bond)
21A	H131/Nε2	2.92 (hydrogen bond)	21B	E97/Cδ	3.21
21B	H131/Cε1	3.23	21B	E97/Oε1	2.84
21B	H131/Nε2	3.35 (hydrogen bond)	21B	E97/Oε2	3.00
22A	R130/Nη2	3.39	21B	Q100/Oε1	2.56
24B	W168/Cζ2	3.29	22A	W168/Cζ2	3.43
25B	W168/Cζ2	3.21	22B	Q100/Nε2	3.45
26B	W168/Cζ2	3.29	22B	Q100/Oε1	3.15
27B	W168/Cζ2	3.45	25A	W168/Cζ2	3.46
28B	W168/Nε1	3.48	31B	Q100/Nε2	3.41
29B	W168/Cζ2	3.43	32A	R130/Nη2	3.31 (hydrogen bond)
29B	W168/Nε1	3.35	32B	H131/Nε2	3.15 (hydrogen bond)
30B	W168/Nε1	3.42			
32B	L104/Cβ	3.30			

*There are two conformations of (2Z,2’Z)-2,2’-disulfanediylbis(3-(6-bromoindol-3-yl)acrylic acid) in the structure, the conformations are labelled as A and B

R.M.S.D. Calculations

Total Protein Calculations

The r. m. s. d. values were calculated for *holo* PEF(S)/PEF(S)-**3** (Fig S1), *holo* PEF(S)/PEF(S)-**4** (Fig. S2) and PEF(S)-3/ PEF(S)-4 (Fig. S3) with Superpose.[12] The values for both the main chain Cα and the side chains were calculated for both chain A and chain B in the asymmetric unit.

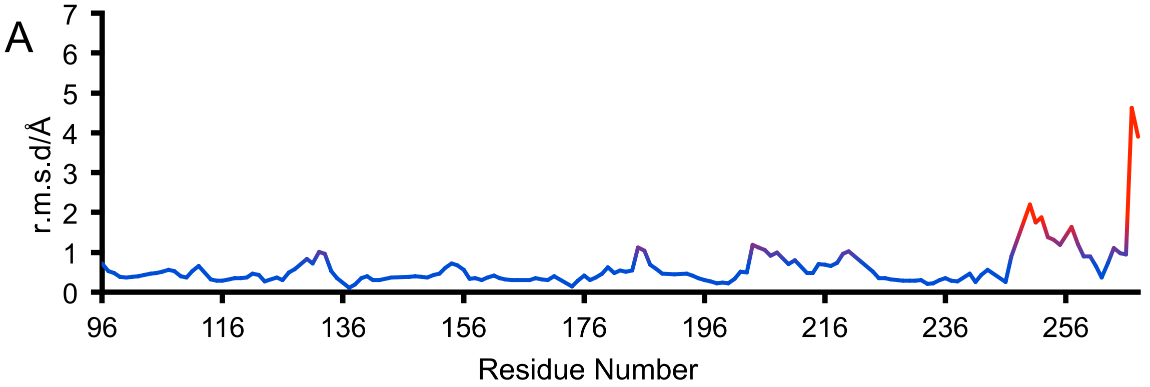

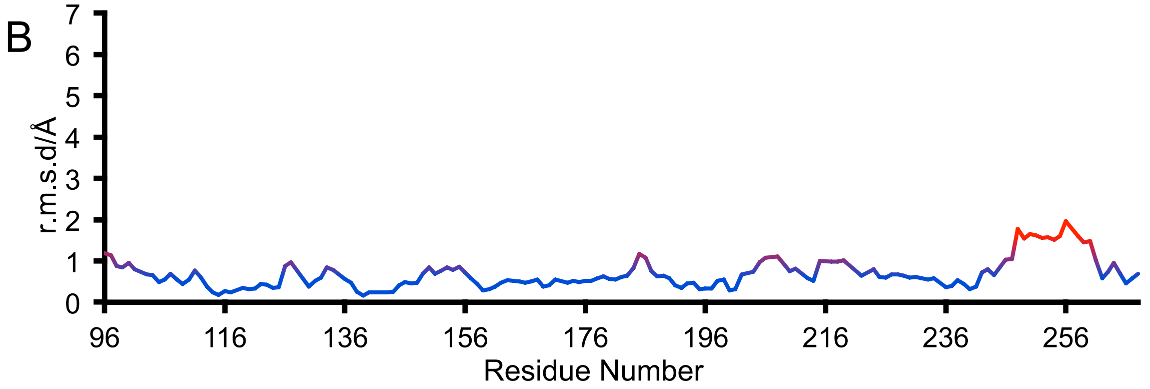

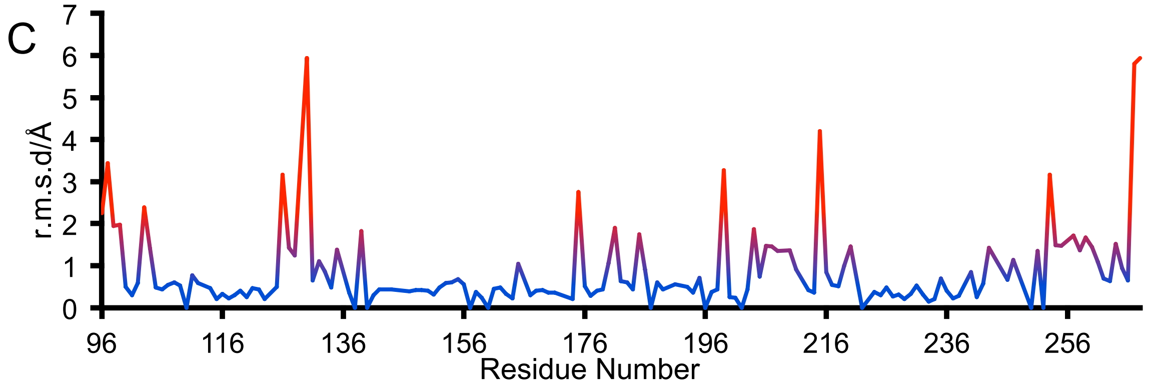

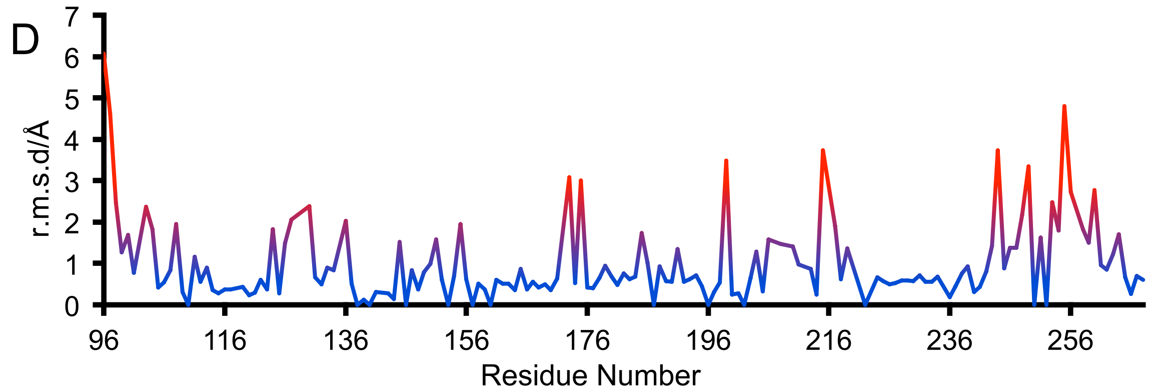

Figure S1: R.m.s.d. values calculated between the Cα's of *holo* PEF(S) (PDB 4PHJ) and PEF(S)-**3** (PDB 4WQ2), (A) chain A and (B) chain B. R.m.s.d. values calculated between the side chains of *holo* PEF(S) and PEF(S)-**3**, (C) chain A and (D) chain B.

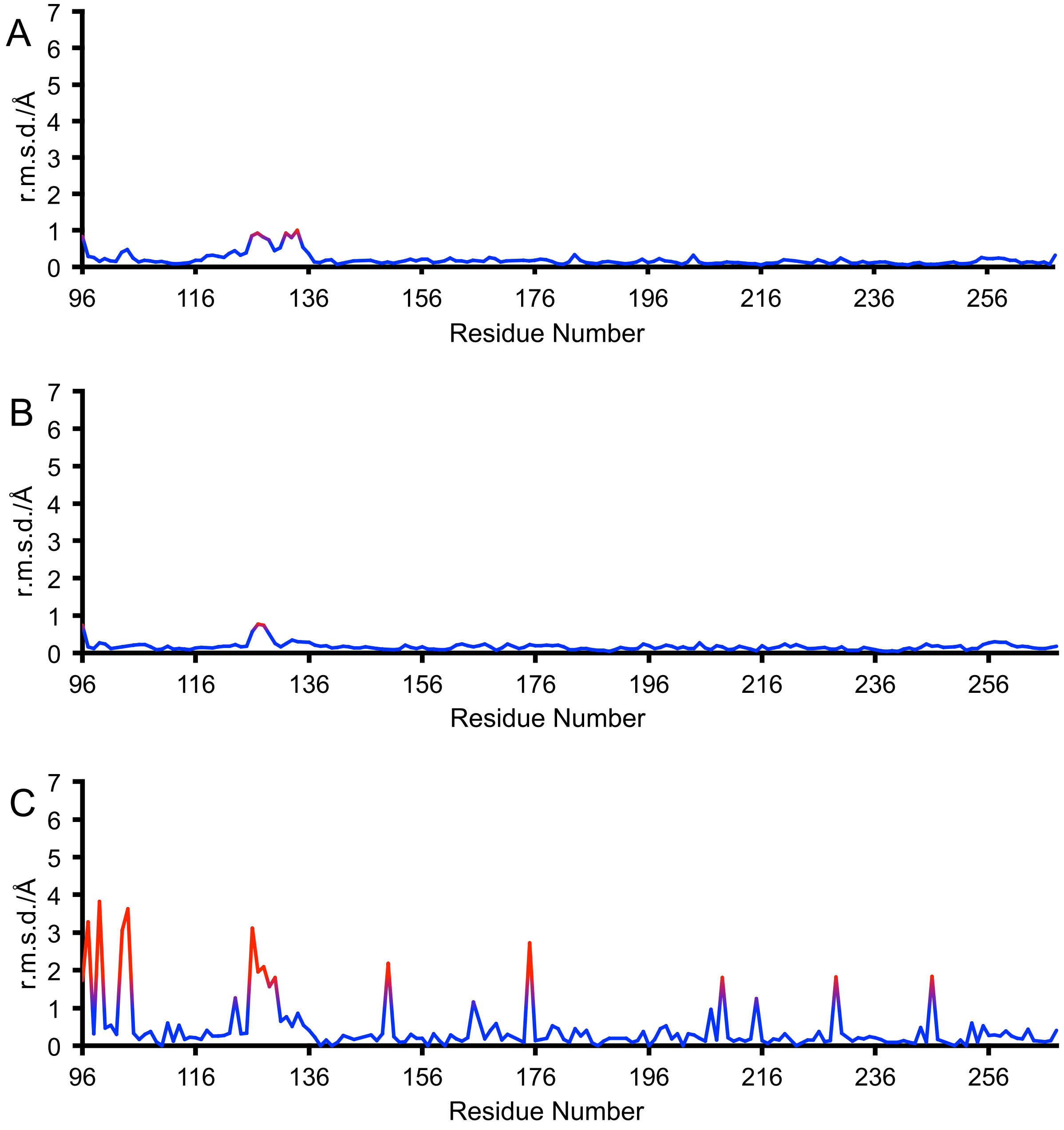

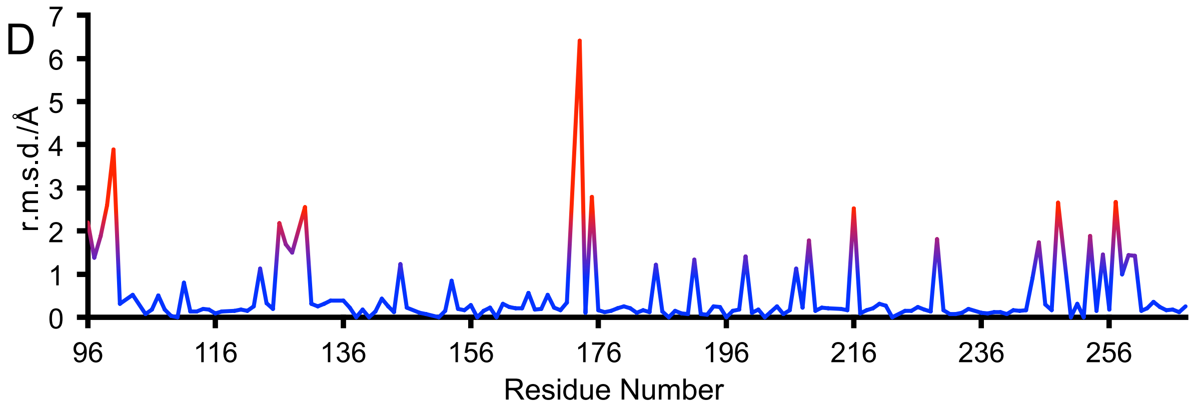

Figure S2: R.m.s.d. values calculated between the Cα's of *holo* PEF(S) (PDB 4PHJ) and PEF(S)-**4** (PDB 4WQ3), (A) chain A and (B) chain B. R.m.s.d. values calculated between the side chains of *holo* PEF(S) and PEF(S)-**3**, (C) chain A and (D) chain B.

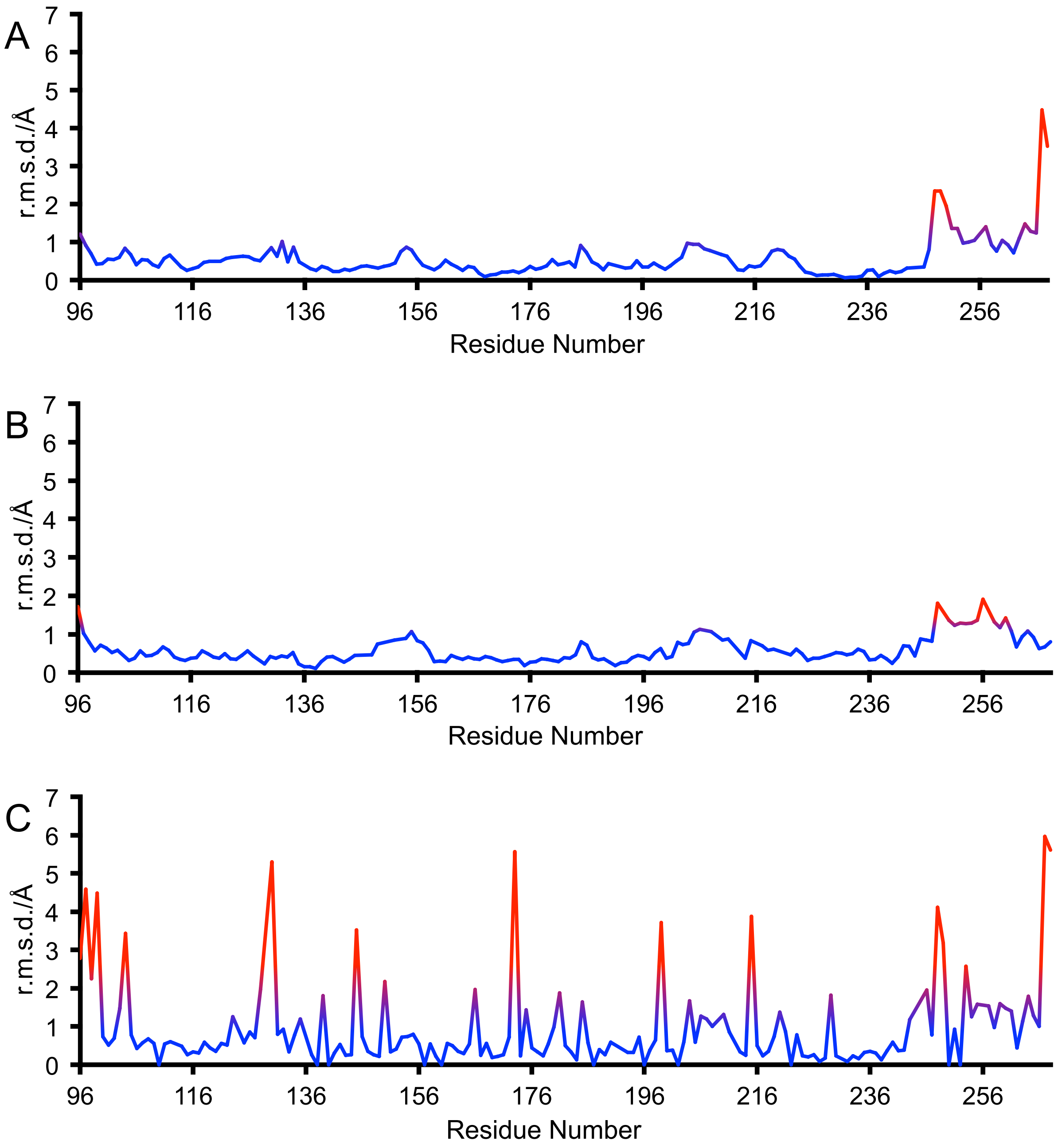

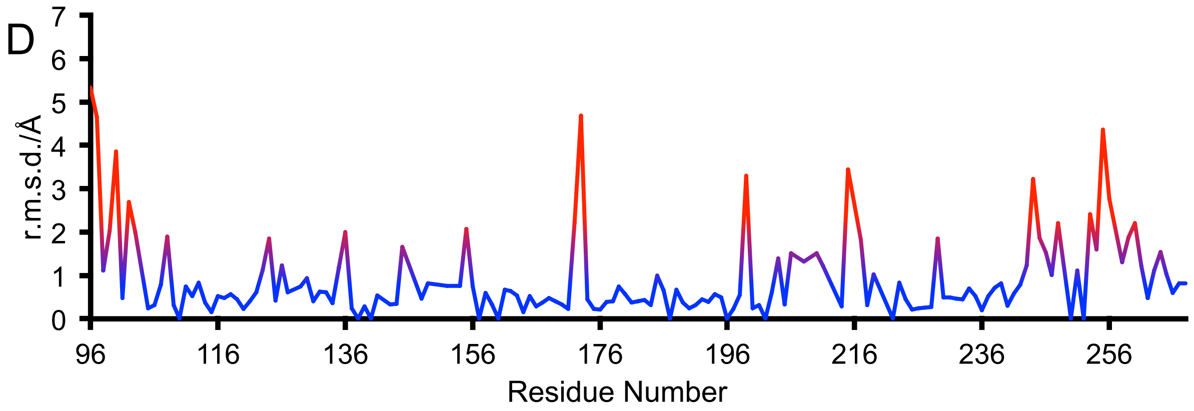

Figure S3: R.m.s.d. values calculated between the Cα's of PEF(S)-**3** (PDB 4WQ2) and PEF(S)-**4** (PDB 4WQ3), (A) chain A and (B) chain B. R.m.s.d. values calculated between the side chains of PEF(S)-**3** and PEF(S)-**4**, (C) chain A and (D) chain B.

Binding Pocket Calculations

The r.m.s.d. values calculated between the inhibitor binding pocket of PEF(S)-**3** (PDB 4WQ2) and *holo*-PEF(S) (PDB 4PHJ).

	**Chain A**		**Chain B**	
**Residue**	*Main chain*	*Side chain*	*Main chain*	*Side chain*
100	0.37	0.50	0.96	1.69
104	0.47	1.42	n/a	n/a
127	0.50	1.43	0.97	2.06
128	0.58	1.25	n/a	n/a
130	0.84	5.94	0.38	2.39
131	0.72	0.65	0.52	0.66
168	0.36	0.41	0.54	0.42
172	n/a	n/a	n/a	n/a
175	0.29	2.75	0.49	3.00

The r.m.s.d. values calculated between the inhibitor binding pockets of PEF(S)-**4** (PDB 4WQ3) and *holo*-PEF(S) (PDB 4PHJ).

	**Chain A**		**Chain B**	
**Residue**	*Main chain*	*Side chain*	*Main chain*	*Side chain*
100	0.23	0.46	0.25	3.89
104	0.47	3.63	n/a	n/a
127	0.92	1.96	0.77	1.69
128	0.81	2.09	0.75	1.51
130	0.45	1.80	0.26	2.56
131	0.53	0.65	0.16	0.31
168	0.26	0.41	0.17	0.52
172	n/a	n/a	n/a	n/a
175	0.16	2.73	0.22	2.80

The r.m.s.d. values calculated between the inhibitor binding pockets of PEF(S)-**3** (PDB 4WQ2) and PEF(S)-**4** (PDB 4WQ3)

	Chain A		Chain B	
**Residue**	*Main Chain*	*Side chain*	*Main Chain*	*Side chain*
100	0.44	0.73	0.71	3.86
104	0.85	3.43	n/a	n/a
127	0.54	0.70	0.44	0.62
128	0.51	1.99	n/a	n/a
130	0.85	5.29	0.42	0.94
131	0.62	0.80	0.38	0.41
168	0.10	0.55	0.42	0.49
172	0.21	0.73	0.32	2.22
175	0.26	1.43	0.18	0.23

Compound Oxidation Analysis

UV-Vis spectrophotometry

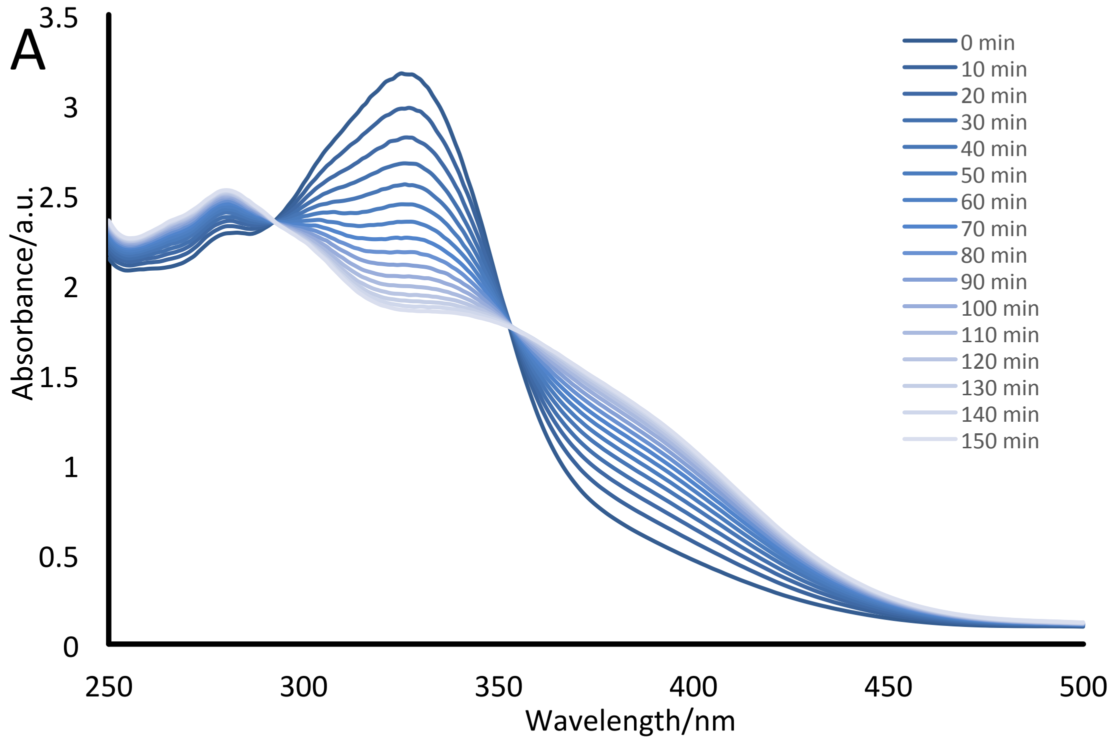

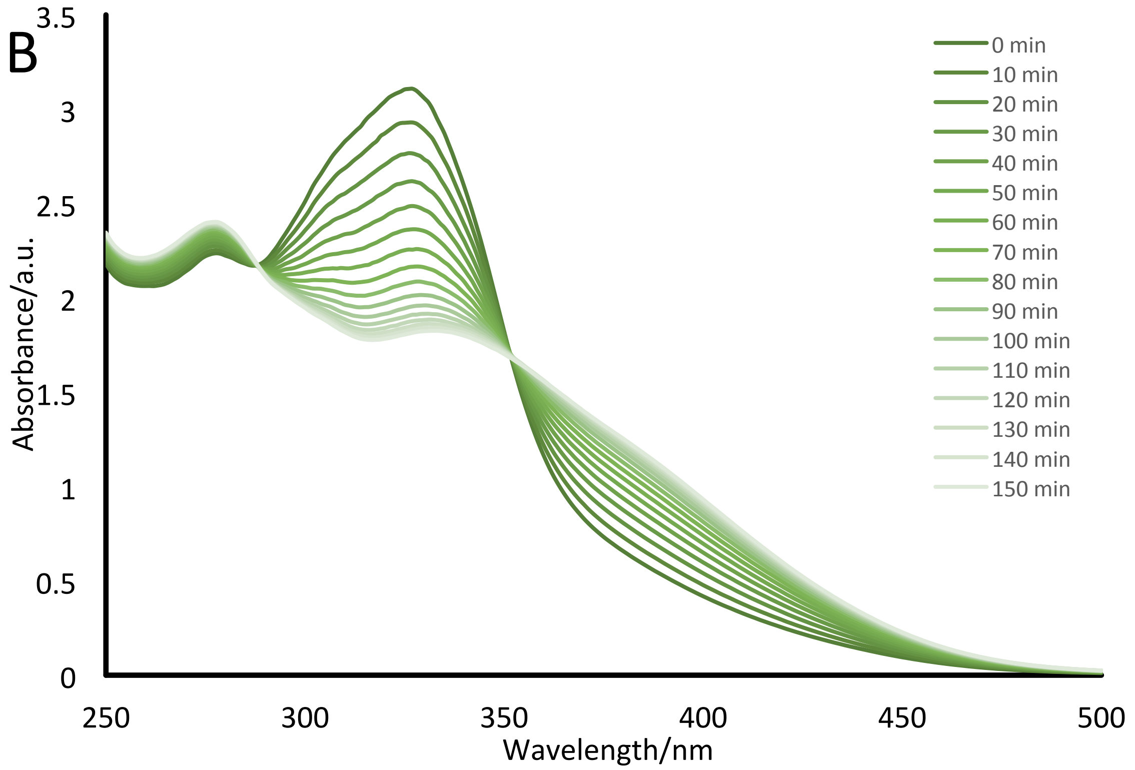

Figure S4: UV-Vis spectrophotometry analysis of (A) (*Z*)-3-(indol-3-yl)-2-mercaptoacrylic acid (250 μM) and (B) (*Z*)-3-(7-chloroindol-3-yl)-2-mercaptoacrylic acid (250 μM) (B) in 10 mM phosphate buffer (pH 7.0) (1 cm^3). Spectra were measured every 10 min for 2.5 hours, a clear spectroscopic change corresponding to formation of the disulfide can be seen in the compound observed by the loss of the λ_{max} = 325 nm (A) and 329 nm (B).

HPLC analysis

(Z)-3-(Indol-3-yl)-2-mercaptoacrylic acid (5 mg) was dissolved in a 1:1 mixture of acetonitrile and water containing 0.1 % trifluoroacetic acid (pH 4.0, 1 mL). This solution (20 μL) was injected onto an analytical C18 reverse phase HPLC column and eluted (detecting at 210 nm, flow rate 1 mL/min, retention time 23 min) with a linear gradient ranging from 9:1 H_2O:acetonitrile (0.1 % TFA) to 100 % acetonitrile (0.1 % TFA) over 50 min. The solution was left for 24 h and was then reinjected. The resulting disulfide eluted with a longer retention time of 24.5 min. Following this a grain (~10 mg) of tris(2-carboxyethyl)phosphine (TCEP) was added and to the solution was reinjected (20 μL) onto the column at hourly intervals. Within 2 h the original sulfhydryl peak reappeared returning to a retention time of 23 min.

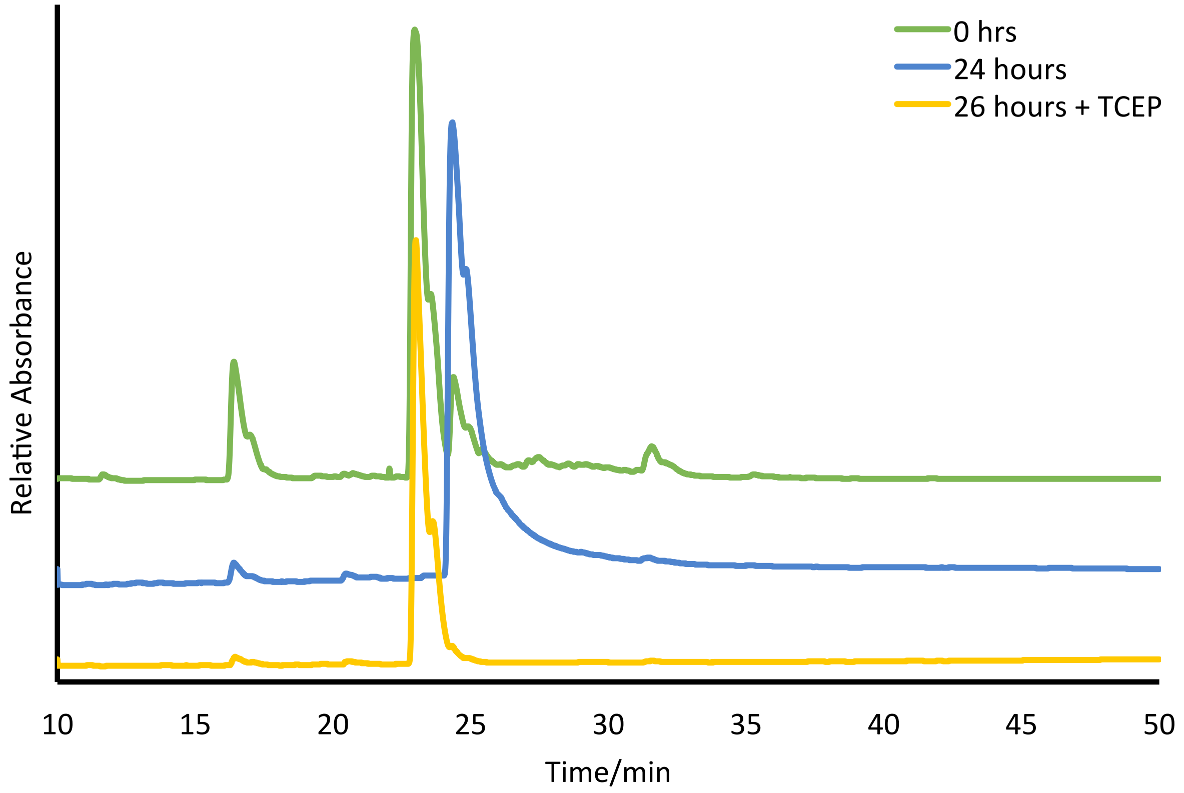

Figure S5: High performance liquid chromatograms (detecting at 210 nm) of the reversible disulfide formation from (*Z*)-3-(indol-3-yl)-2-mercaptoacrylic acid through aerial oxidation followed by reduction with TCEP.

(Z)-3-(6-Bromoindol-3-yl)-2-mercaptoacrylic acid (5 mg) was dissolved in a 1:1 mixture of acetonitrile and water containing 0.1 % trifluoroacetic acid (pH 4.0, 1 mL). This solution (20 μL) was injected onto an analytical C18 reverse phase HPLC column and eluted (detecting at 210 nm, flow rate 1 mL/min, retention time 27 min) with a linear gradient ranging from 9:1 H_2O:acetonitrile (0.1 % TFA) to 100 % acetonitrile (0.1 % TFA) over 50 min. The solution was left for 24 hr and was then reinjected. The resulting disulfide eluted with a longer retention time of 30 min.

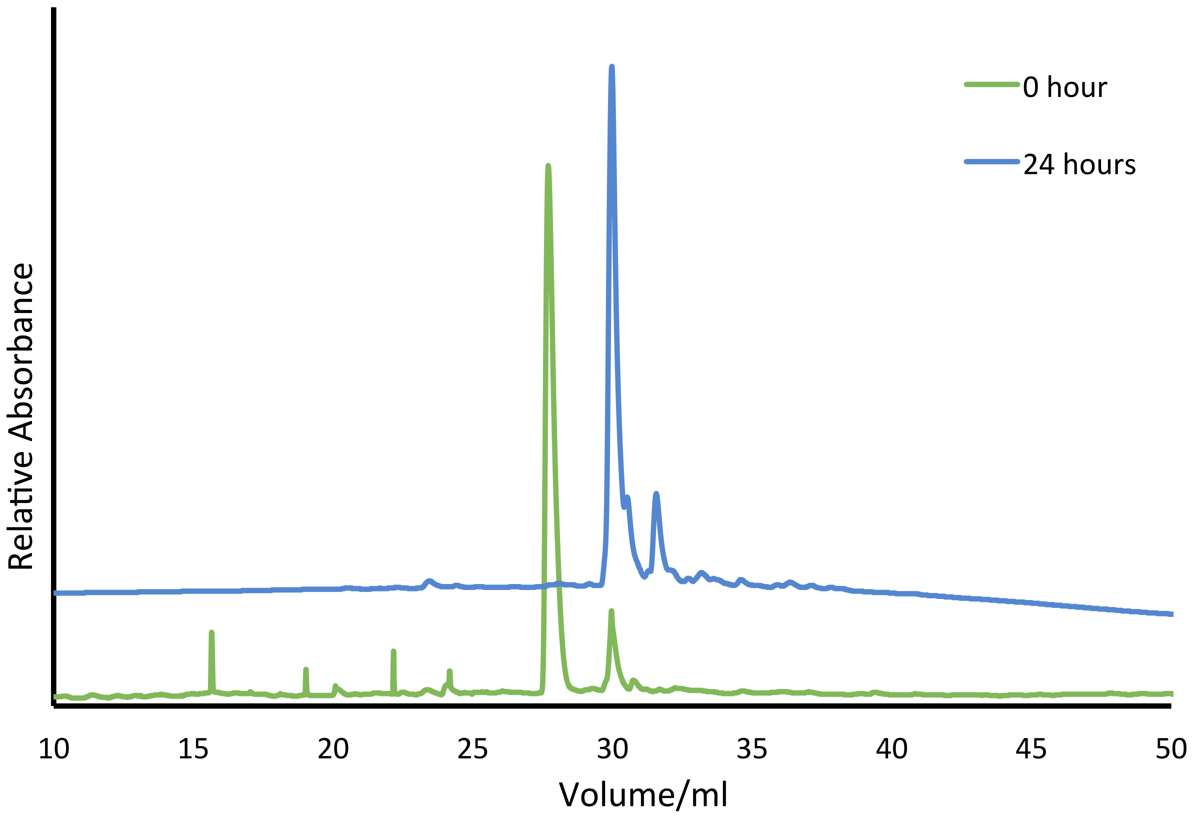

Figure S6: High performance liquid chromatograms (detecting at 210 nm) of the disulfide formation of (2Z,2'Z)-2,2'-disulfanediylbis(3-(6-bromoindol-3-yl)acrylic acid) (**4**) from (*Z*)-3-(6-bromoindol-3-yl)-2-mercaptoacrylics acid (**3**) through aerial oxidation.

NMR Spectroscopic Analysis

(*Z*)-3-(6-Bromoindol-3-yl)-2-mercaptoacrylic acid (**3**, 5 mg) was dissolved in δ_6-DMSO and a ^{1}H NMR spectrum (400 MHz) was recorded immediately, the sample was left to stand for 10 h whereupon another ^{1}H NMR spectrum was recorded. The signals corresponding to N-*H*, *H*C=CS and *H*C-NH all shift; δ_H 11.90 to 12.06, 8.00 to 8.15 and 7.99 to 8.41, respectively. A third spectrum was recorded after 24 h. The signals corresponding to N-*H*, *H*C=CS and *H*C-NH in **3** had disappeared and the disulfide compound, (2Z,2'Z)-2,2'-disulfanediylbis(3-(6-bromoindol-3-yl)acrylic acid) (**4**), had fully formed with the signals corresponding to these protons at d_H 12.06, 8.15 and 8.41 respectively.

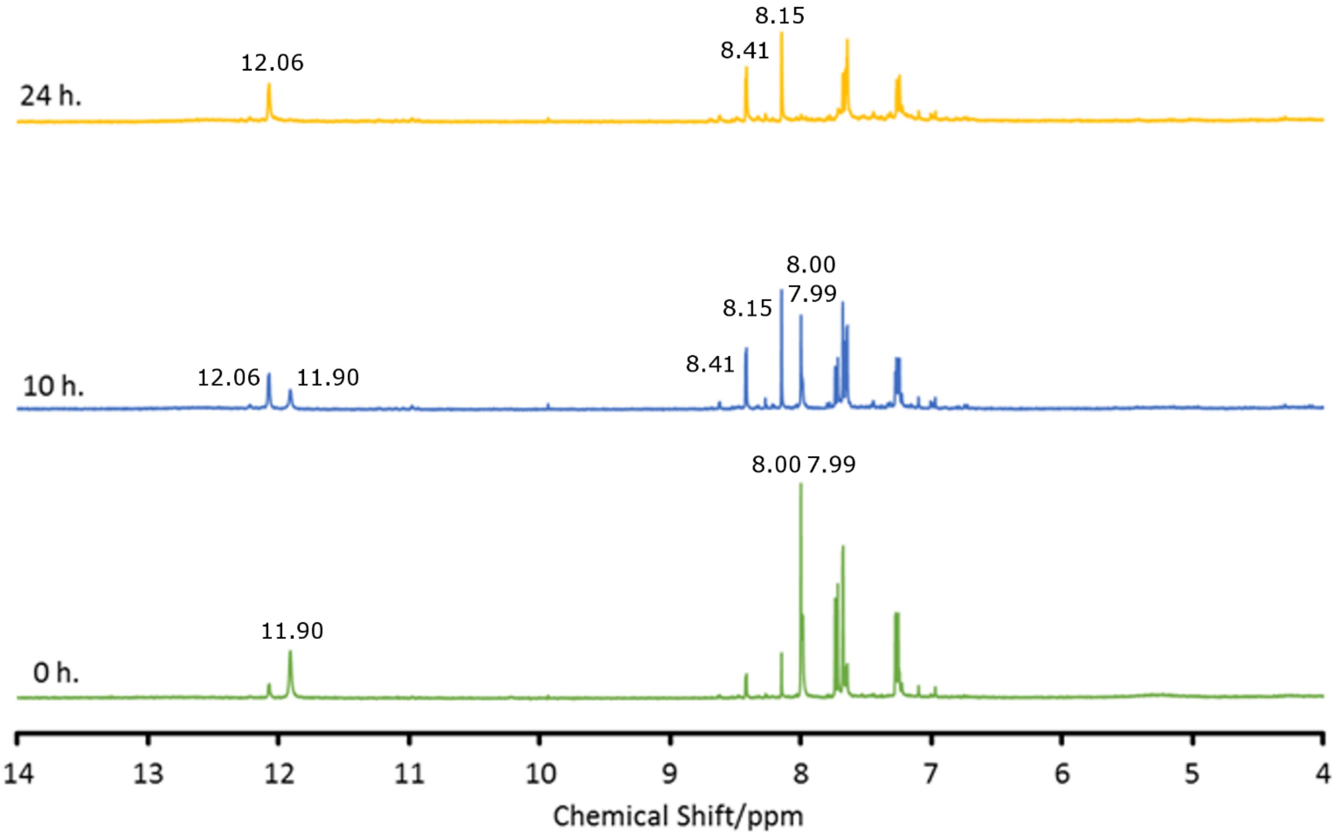

Figure S7: ^{1}H NMR (400 MHz, d6-DMSO) spectra showing formation of the disulfide (2Z,2'Z)-2,2'-disulfanediylbis(3-(6-bromoindol-3-yl)acrylic acid) (**4**) from (*Z*)-3-(6-bromoindol-3-yl)-2-mercaptoacrylic acid (**3**) through aerial oxidation.

Organic Synthesis

General synthetic procedures and the synthesis of (*Z*)-3-(6-bromoindol-3-yl)-2-mercaptoacrylic acid and the other mercaptoacrylic acid derivatives were as described previously.[13]

Oxidation reaction

(2Z,2'Z)-2,2'-disulfanediylbis(3-(6-bromoindol-3-yl)acrylic acid)[14]

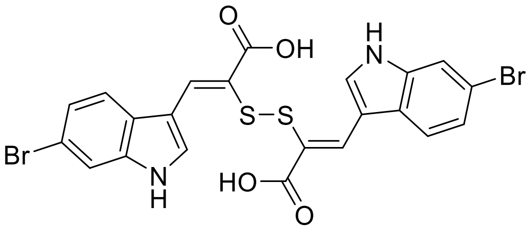

A reaction mixture containing (*Z*)-3-(6-bromoindol-3-yl)-2-mercaptoacrylic acid (51 mg, 0.17 mmol), triethylamine (50 µl) and iodine (10 mg, 0.04 mmol) in acetonitrile (2 ml) was left to stir at 25 °C for 1.5 hours. 2 M HCl (5 ml) was used to quench the reaction mixture. The precipitate that formed was filtered and washed with water and ether and air dried to form a light brown solid in a 65% yield (33 mg, 55 µmol). δ_H (600 MHz, DMSO): 12.06 (2 H, s, NH) 8.38 (2 H, s , ArCH), 8.10 (2 H, s, ArCH), 7.66 (2 H, d, *J* = 8.9, ArCH), 7.64 (2 H, s, ArCH), 7.25 (2 H, d, *J* = 8.7, ArCH). δ_C (150 MHz, DMSO): 167.4 (*C*OOH), 138.0 (C=*C*H), 136.7 (ArC), 131.5 (ArCH), 127.1 (ArC), 124.1 (ArCH), 121.4 (ArCBr), 120.3 (ArCH), 115.6 (*C*=CH), 115.3 (ArCH), 110.9 (ArC). Mass spectrum: HRMS (ES^-) found 590.8674, $C_{22}H_{13}N_2O_4S_2Br_2$ calculated 590.8683.

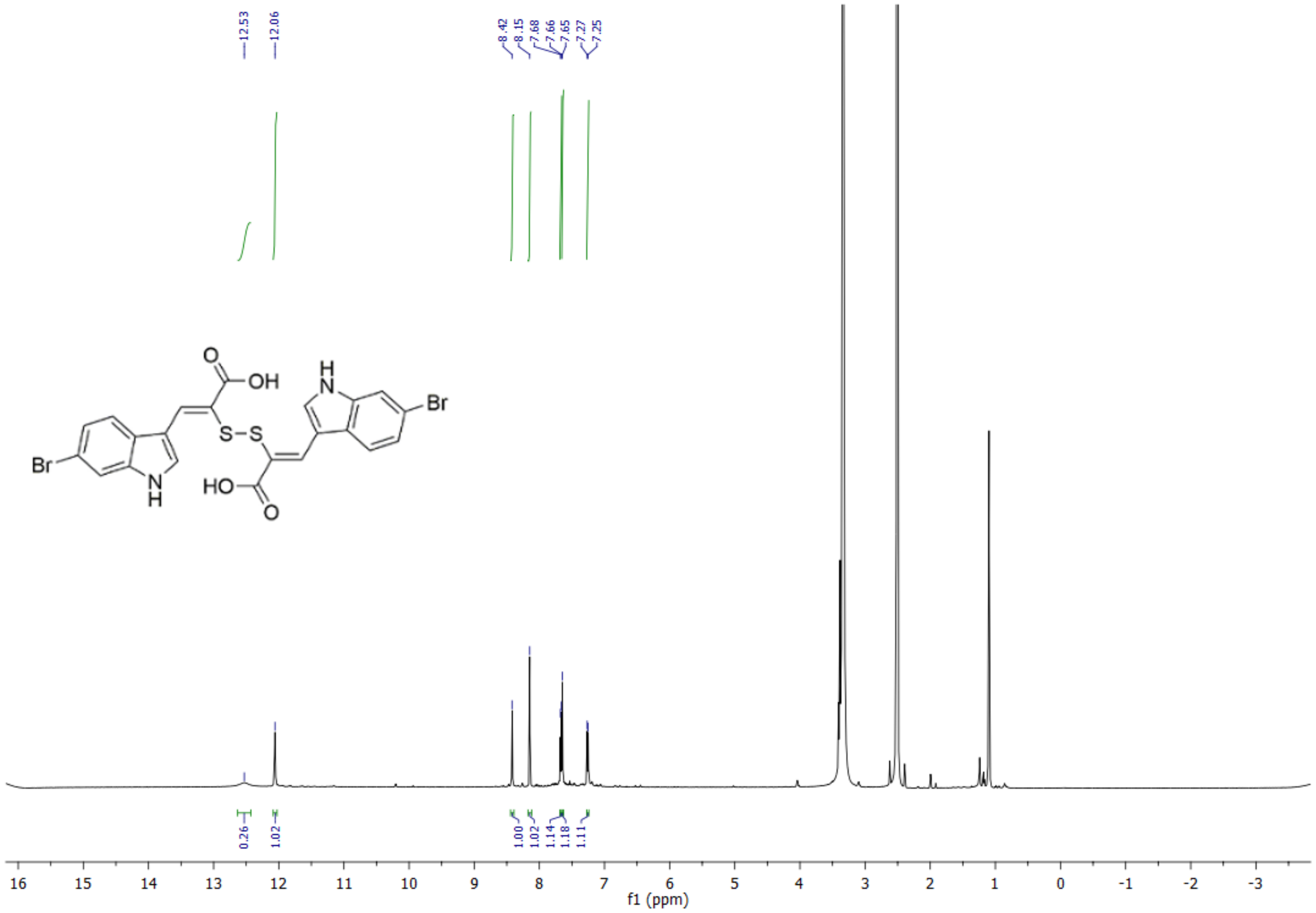

Thioether synthesis

Baylis Hillman reaction

Methyl 2-((4-bromophenyl)(hydroxyl)methyl)acrylate

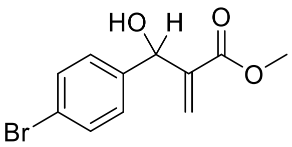

A solution of 4-bromobenzaldehyde (1.00 g, 5.42 mmol), methyl acrylate (0.50 mL, 5.52 mmol) and DABCO (0.37 g, 3.30 mmol) in methanol (10 mL) was left to stir at room temperature (20 °C) for 72 hours. The reaction was quenched with 1 M HCl (10 mL) and the compound was extracted with diethyl ether (2 × 50 mL). The organic layer was pooled, washed with brine and dried over $MgSO_4$ and concentrated under reduced pressure. Flash chromatography with a solvent mixture of 9:1 of hexane/ethyl acetate was used to obtain the title compound as a white solid (1.00g, 69%). m.p. 65 - 68 °C. δ_H (300 MHz, $CDCl_3$): 7.48 (2 H, d, *J* = 8.5, ArCH), 7.28 (2 H, d, *J* = 8.5, ArCH), 6.36 (1 H, s, C=C*H*H), 5.84 (1H, s, C=CH*H*), 5.53 (1 H, d, *J* = 5.5, C*H*OH), 3.75 (3H, s, C*H*$_3$), 3.09 (1 H, d, *J* = 5.8, C*H*OH). δ_C (75 MHz, $CDCl_3$): 166.7 (*C*OOCH$_3$), 141.4 (ArC), 140.3 (ArC), 131.6 (2 ArCH), 128.3 (2 ArCH), 126.6 (C=*C*H$_2$), 121.8 (ArCBr), 72.9 (H*C*OH), 52.0 (COO*C*H$_3$). Mass spectrum: HRMS (EI^+) found 269.9892, $C_{11}H_{11}O_3Br$ calculated 269.9892

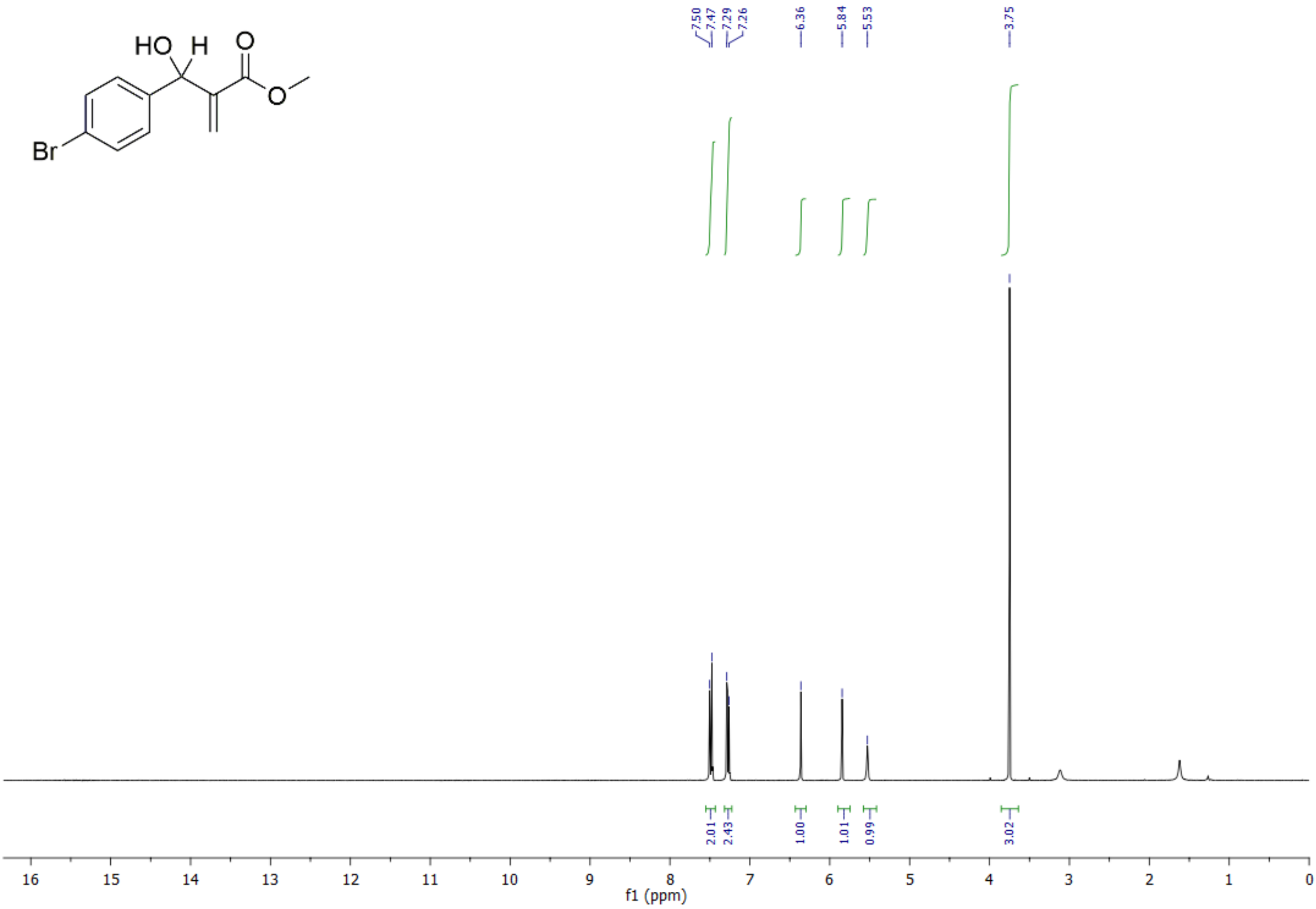

Methyl 2-((3-chlorophenyl)(hydroxyl)methyl)acrylate

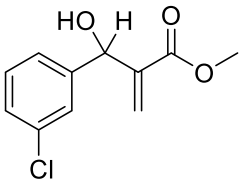

Appearance; pale yellow oil. Yield: 68%. δ_H (300 MHz, $CDCl_3$): 7.30 (2 H, d, *J* = 3.0, ArCH), 7.29 (2 H, d, *J* = 3.0, ArCH), 6.38 (1 H, s, C=C*H*H), 5.86 (1H, s, C=CH*H*), 5.54 (1 H, d, *J* = 6.2, C*H*OH), 3.76 (3H, s, C*H*$_3$), 3.12 (1 H, d, *J* = 6.0, C*H*OH). δ_C (75 MHz, $CDCl_3$): 166.6 (*C*OOCH$_3$), 143.3 (Ar*C*CHOH), 141.3 (*C*=CH$_2$), 134.4 (ArCCl), 129.7 (ArCH), 128.0 (ArC), 126.8 (C=*C*H$_2$), 124.8 (ArCH), 72.8 (H*C*OH), 52.2 (COO*C*H$_3$). Mass spectrum: HRMS (EI^+) found 226.0388, $C_{11}H_{11}O_3Cl$ calculated 226.0397

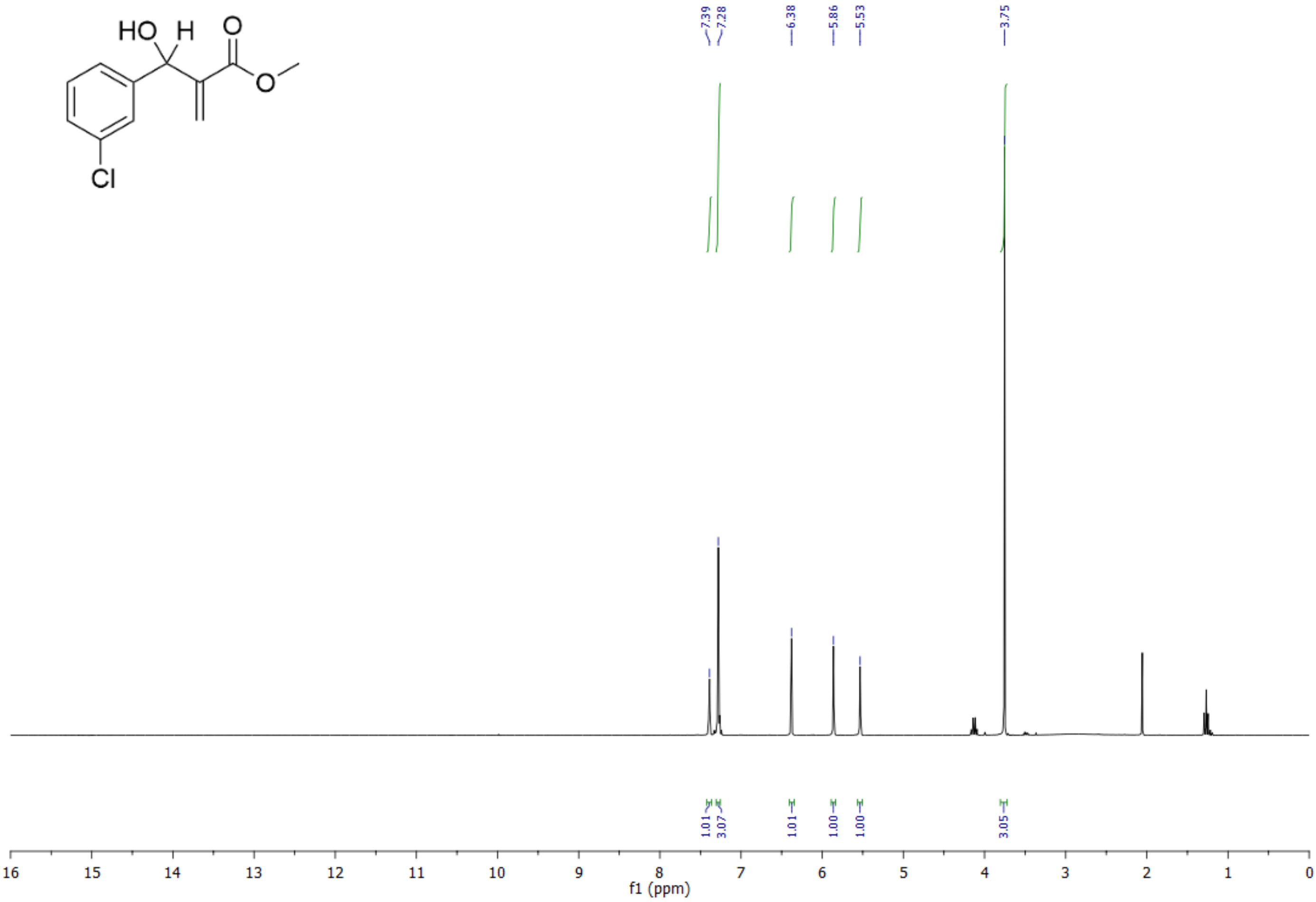

Hydrolysis of the methyl ester

2-((4-bromophenyl)(hydroxyl)methyl)acrylate

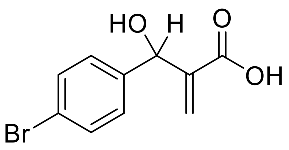

A suspension of methyl 2-((4-bromophenyl)(hydroxyl)methyl)acrylate (0.3 g, 1.10 mmol) in methanol (4 mL) and 2 M NaOH (4 mL) was stirred for 16 h at room temperature. The reaction mixture was quenched with 2 M HCl (10 mL) and extracted with diethyl ether (2 × 15 mL). The organic layer was washed with brine (30 mL) and dried over anhydrous $MgSO_4$ followed by filtration under reduced pressure. The solution was concentrated under reduced pressure and was carried on to the next reaction without further purification (0.28 g). m.p. 74 - 96 °C. δ_H (300 MHz, $CDCl_3$): 7.42 (2 H, d, J = 6.5, ArCH), 7.19 (2 H, d, J = 6.5, ArCH), 6.42 (1 H, s, C=C*H*H), 5.89 (1H, s, C=CH*H*), 5.23 (1 H, s, C*H*OH). δ_C (75 MHz, $CDCl_3$): 170.7(*C*OOH), 140.9 (ArC), 139.9 (ArC), 131.7 (2 ArCH), 129.0 (C=*C*H_2), 128.4 (2 ArCH), 122.0 (ArCBr), 72.3 (*C*HOH). Mass spectrum: HRMS (AP$^-$) found 254.9652, $C_{10}H_8O_3{}^{79}Br$ calculated 254.9657.

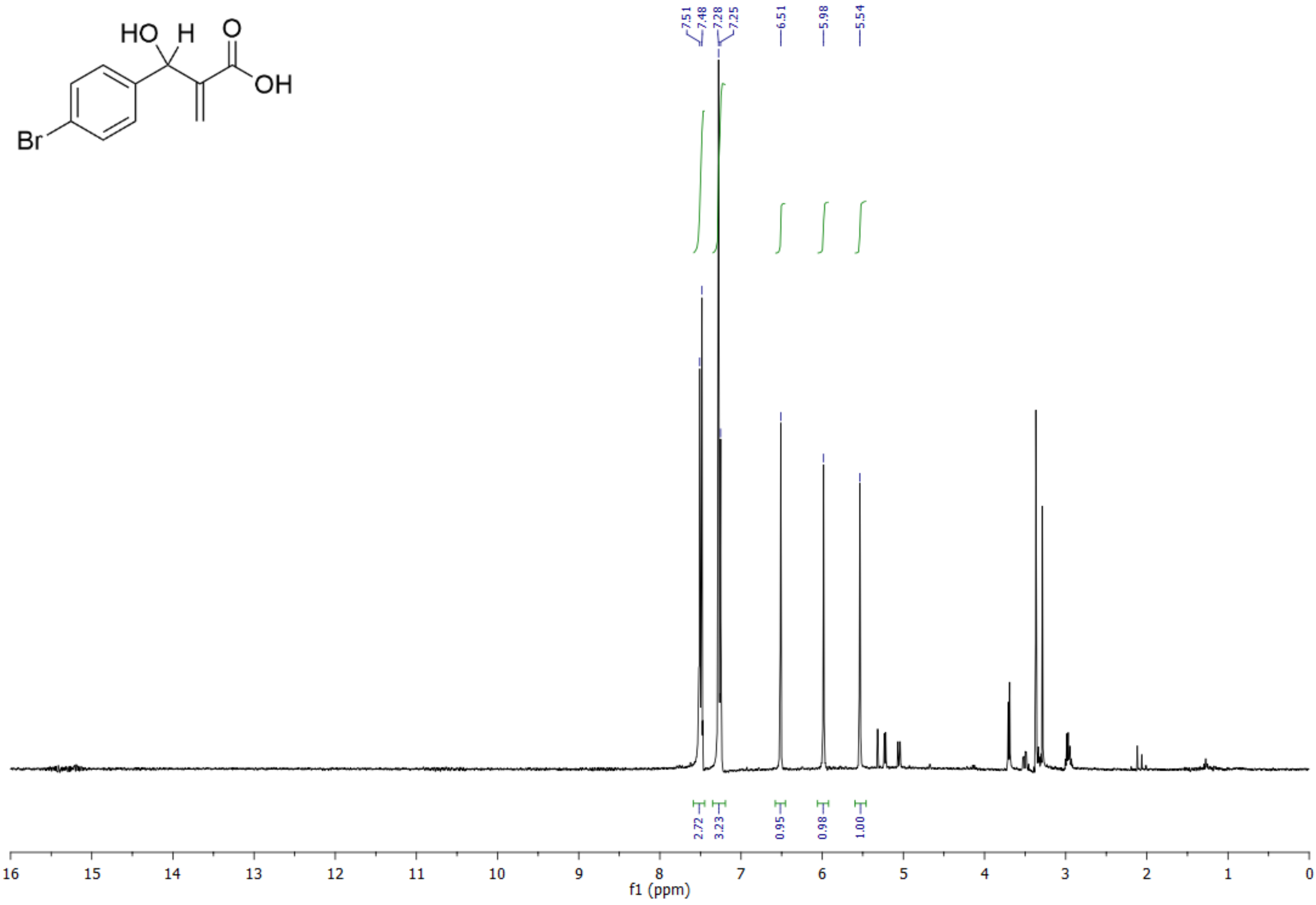

2-((3-Chlorophenyl)(hydroxyl)methyl)acrylate

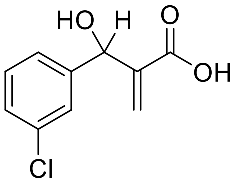

Appearance; pale yellow oil. Crude product. δ_H (300 MHz, $CDCl_3$): 7.30 (2 H, d, J = 3.0, ArCH), 7.29 (2 H, d, J = 3.0, ArCH), 6.38 (1 H, s, C=C*H*H), 5.86 (1H, s, C=CH*H*), 5.54 (1 H, d, J = 6.2, C*H*OH), 3.76 (3H, s, CH_3), 3.12 (1 H, d, J = 6.0, CH*O*H). δ_C (75 MHz, $CDCl_3$): 170.5 (*C*OOH), 143.0 (Ar*C*CHOH), 140.7 (*C*=CH_2), 133.5 (ArCCl), 129.8 (ArCH), 129.2 (C=CH_2), 128.2 (ArCH), 126.8 (ArCH), 124.8 (ArCH), 72.4 (CHOH). Mass spectrum: HRMS (AP^-) found 211.0171, $C_{10}H_8O_3Cl$ calculated 211.0162.

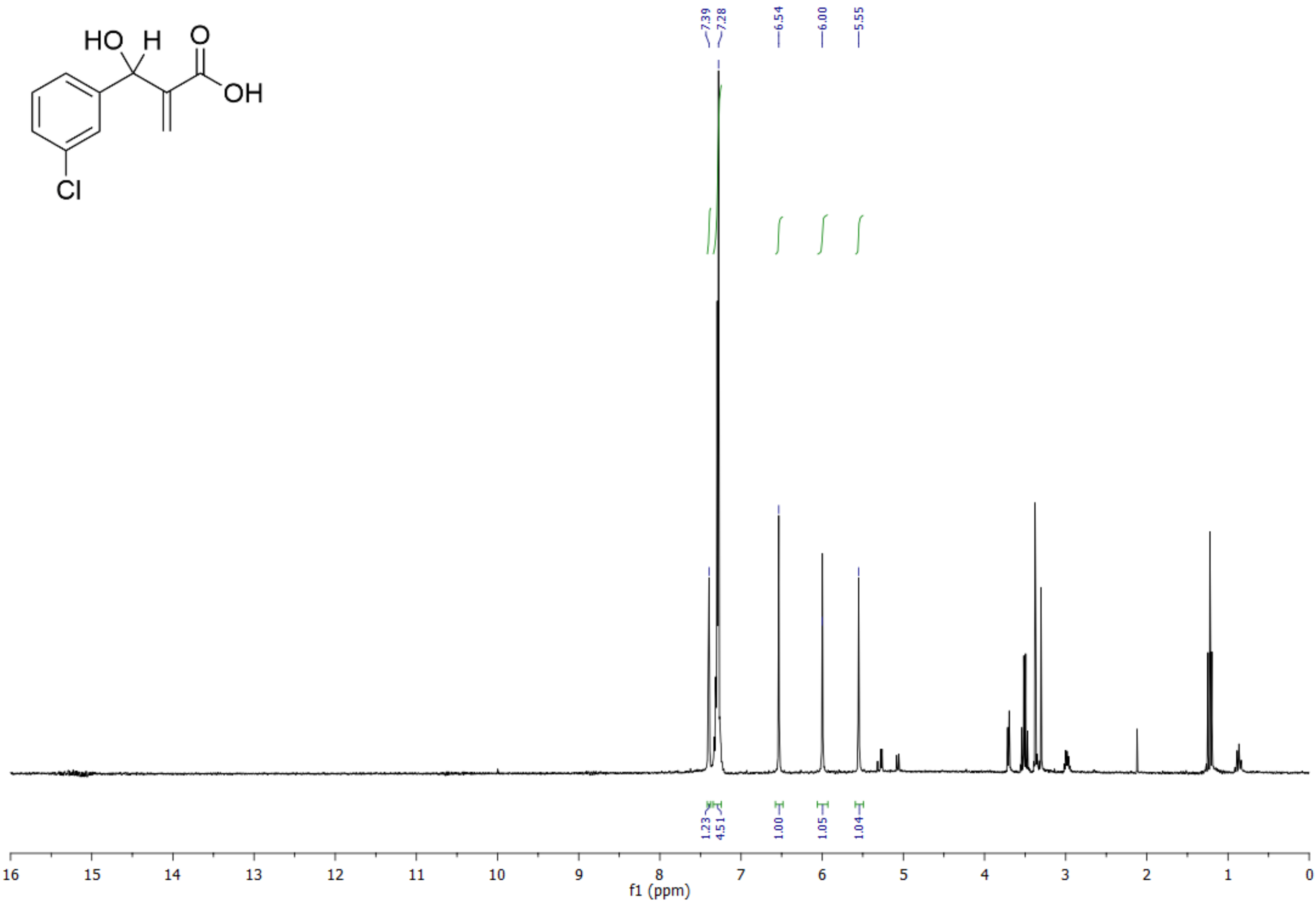

Bromination reaction[15]

(Z)-2-(Bromomethyl)-3-(4-bromophenyl)acrylic acid

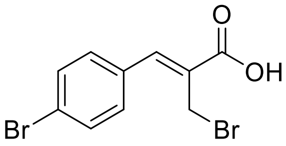

A suspension of 2-((4-bromophenyl)(hydroxyl)methyl)acrylate (0.22 g, 0.87 mmol) in HBr (45% solution in water, 2 mL) and concentrated H_2SO_4 (0.1 mL) was stirred for 16 h at room temperature. Water was added to the reaction mixture and the compound was extracted into diethyl ether (2 × 20 mL). The organic layer was washed with brine (40 mL), dried over anhydrous $MgSO_4$, then filtered and concentrated under reduced pressure to produce the title compound as a white solid which was carried on to the next reaction without further purification (0.26 g). m.p. 155 – 170 °C δ_H (300 MHz, $CDCl_3$): 7.79 (1 H, s, *H*C=C), 7.56 (2 H, d, *J* = 8.5, ArCH), 7.42 (1 H, d, *J* = 8.5, ArCH), 4.29 (2H, s, C*H*$_2$Br). δ_C (75 MHz, $CDCl_3$): 171.1 (*C*OOH), 143.8 (H*C*=CCH_2), 132.8 (ArC), 132.3 (2 ArCH), 131.4 (2 ArCH), 128.4 (ArC), 124.7 (ArCBr), 25.6 (CH_2). Mass spectrum: HRMS (EI^+) found 317.8875 $C_{10}H_8O_2Br_2$ calculated 317.8891.

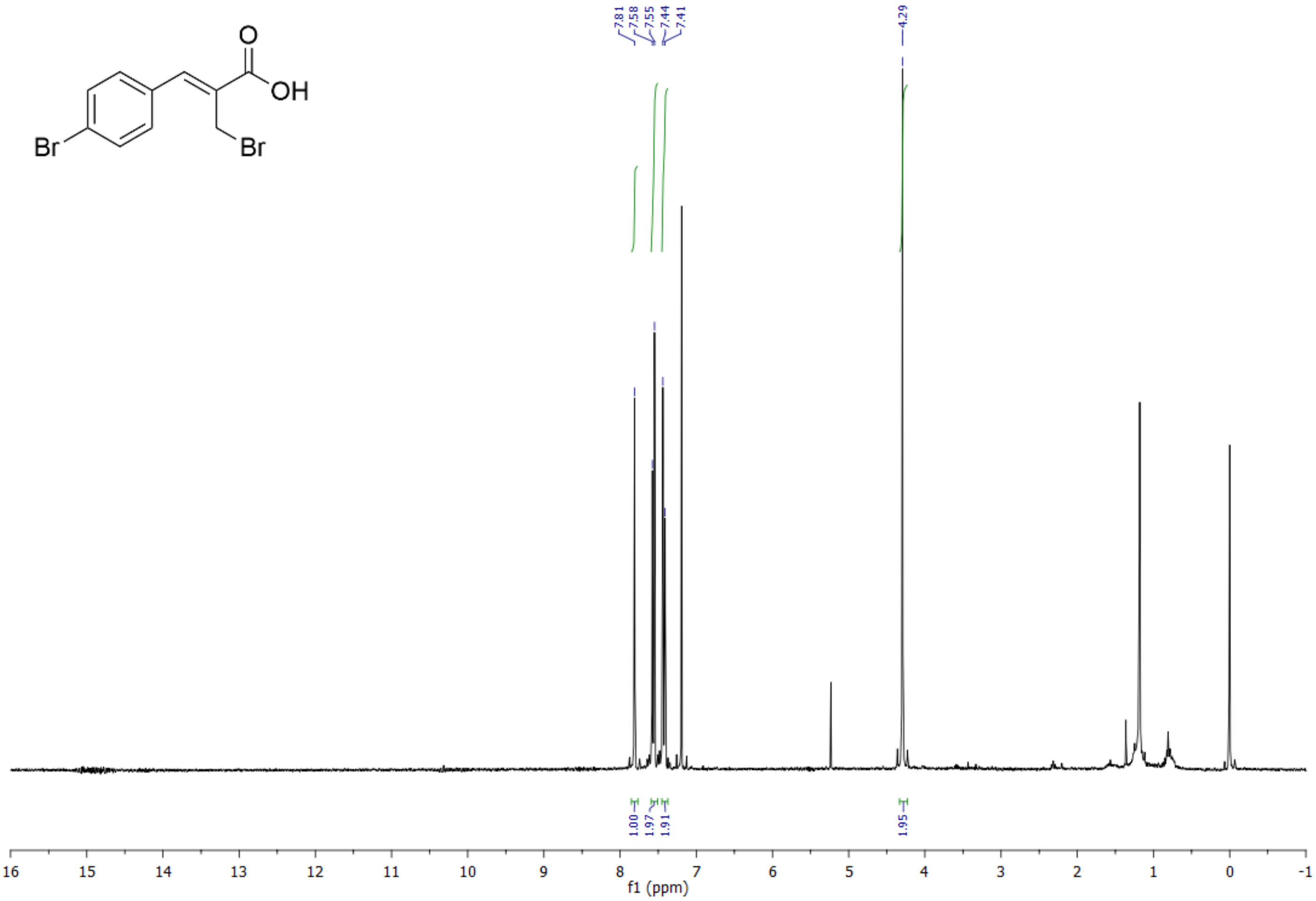

(Z)-2-(Bromomethyl)-3-(3-chlorophenyl)acrylic acid

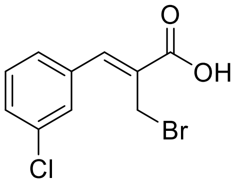

Appearance; white solid. Yield: quanitative. m. p. 136 – 141 °C. δ_H (300 MHz, $CDCl_3$): 7.90 (1 H, s, C*H*=CH_2), 7.61 (1 H, m, ArCH), 7.54 (1 H, m, ArCH), 7.44 (2 H, m, ArCH), 4.38 (1 H, s, CH_2). δ_C (75 MHz, $CDCl_3$): 171.2 (*C*OOH), 143.4 (H*C*=CCH_2), 135.6 (ArC), 135.0 (ArCCl), 130.3 (ArCH), 130.1 (ArCH), 129.7 (ArCH), 129.2 (ArC), 127.7 (ArCH), 25.3 (CH_2). Mass spectrum: HRMS (EI^+) found 273.9387, $C_{10}H_8O_2BrCl$ calculated 273.9396.

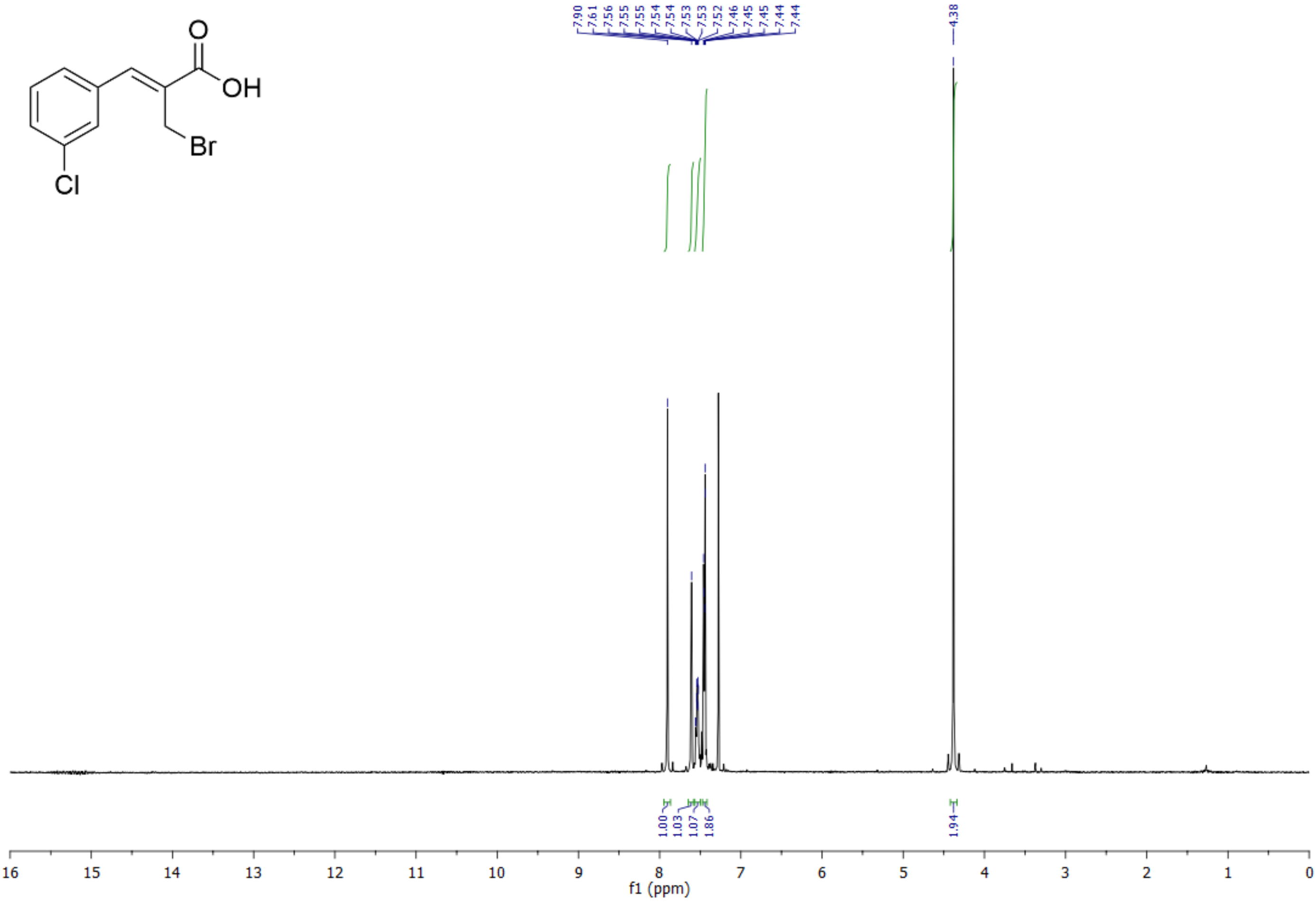

General method for the synthesis of thioether compounds

(Z)-3-(6-Bromoindol-3-yl)-2-(((Z)-3-(4-bromophenyl)-2-carboxyallyl)thio)acrylic acid

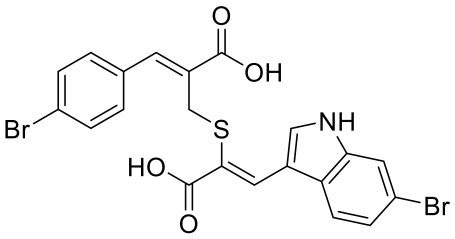

A reaction mixture containing (*Z*)-2-(bromomethyl)-3-(4-bromophenyl)acrylic acid (84 mg, 0.28 mmol), (*Z*)-3-(6-bromoindol-3-yl)-2-mercaptoacrylic acid (84 mg, 0.26 mmol), a catalytic amount of triethylamine (1 drop) in acetonitrile (10 mL) was stirred at room temperature for 16 hours. 1 M HCl (10 mL) was used to acidify the solution and the precipitate that formed was extracted with dichloromethane (2 × 30 mL) and washed with water (2 × 20 mL) and brine (2 × 20 mL). The organic later was then dried over anhydrous $MgSO_4$, filtered and concentrated under reduced pressure. The solid that formed was recrystallized from methanol to yield the title compound as a pale yellow solid (34 mg, 22%). m.p. 230 – 232 °C δ_H (600 MHz, DMSO): 12.75 (2 H, s, COO*H*) 12.01 (1 H, d, *J* = 2.0, NH), 8.57 (1 H, d, *J* = 2.8, ArCH), 8.24 (1 H, s, *H*C=CS), 7.74 (1 H, d, *J* = 8.5, ArCH), 7.67 (1 H, d, *J* = 1.6, ArCH), 7.63 (2 H, d, *J* = 8.5, ArCH), 7.61 (1 H, s, *H*C=CCH$_2$), 7.55 (2 H, d, *J* = 8.5, ArCH), 7.30 (1 H, dd, J_1 = 8.5, J_2 = 2.0, ArCH), 3.90 (2H, s, C*H*$_2$). δ_C (150 MHz, DMSO): 168.5 (*C*OOH), 167.6 (*C*OOH), 139.4 (H*C*=CCH$_2$), 136.9 (H*C*=CS), 134.2 (ArC), 132.4 (2ArCH), 132.0 (2ArCH), 131.3 (ArCH), 130.2 (ArC), 127.1 (ArC), 123.9 (ArCH), 123.0 (ArCBr), 120.2 (ArCH), 119.5 (ArCBr), 115.5 (ArC), 115.2 (ArCH), 110.0 (ArC), 31.6 (*C*H$_2$). Mass spectrum: HRMS (ES$^-$) found 533.9023, $C_{21}H_{14}NO_4SBr_2$ calculated 533.9010.

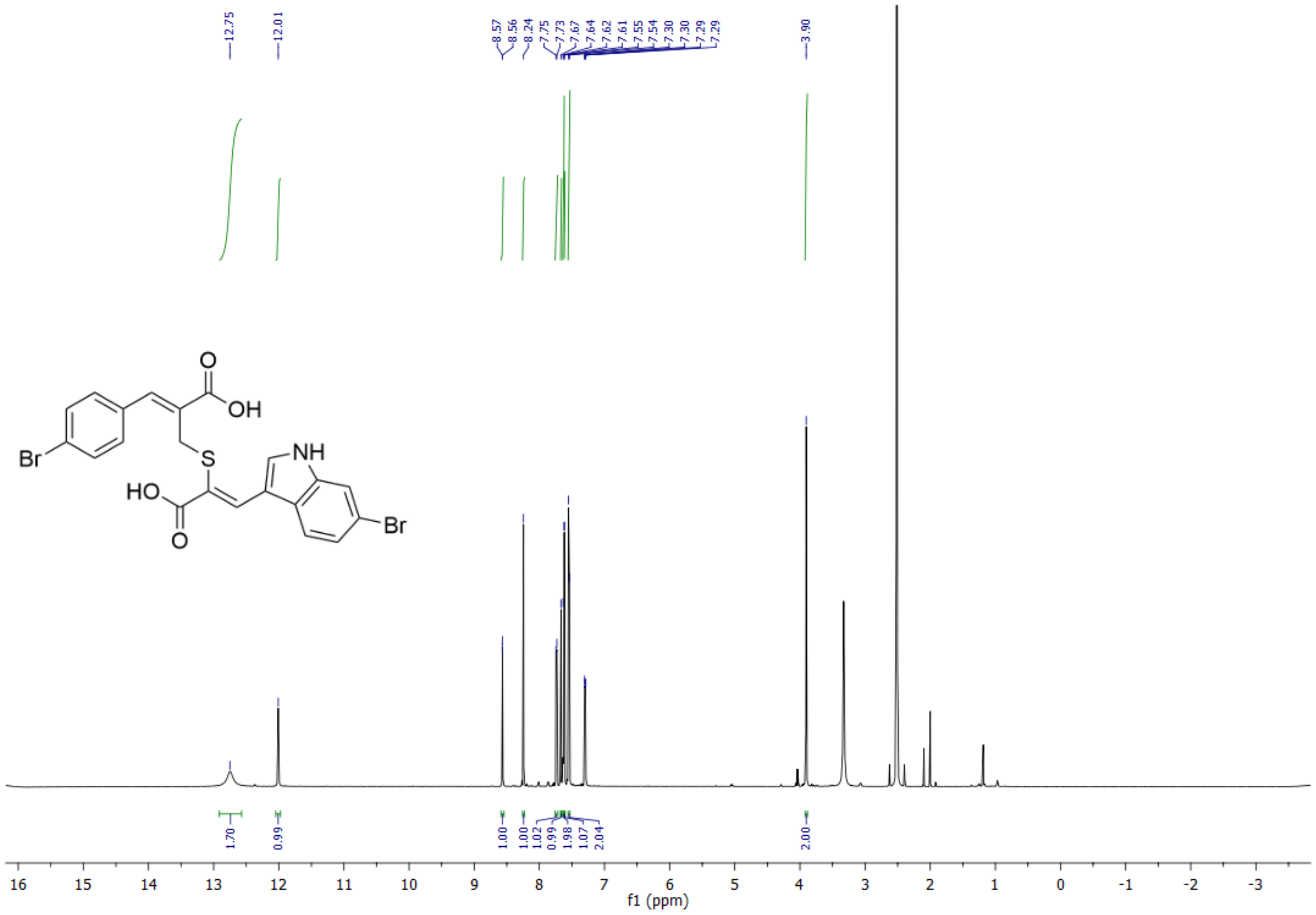

(Z)-3-(4-Bromophenyl)-2-(((Z)-2-(4-bromophenyl)-1-carboxyallyl)thio)acrylic acid

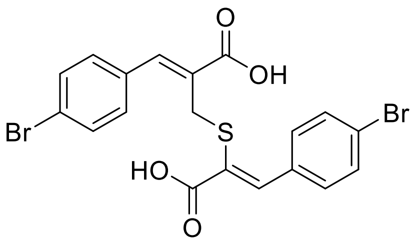

Yield: 39%. m. p. 274 – 277 °C . δ_H (300 MHz, DMSO): 13.06 (2 H, s, COOH), 7.81 (1 H, s, *H*C=CS), 7.78 (2 H, d, *J* = 8.5, ArCH), 7.59 (5 H, m, ArCH and *H*C=CCH$_2$), 7.52 (2 H, d, *J* = 8.5, ArCH), 3.89 (2 H, s, C*H*$_2$). δ_C (150 MHz, DMSO): 168.2 (*C*OOH), 166.8 (*C*OOH), 142.1 (H*C*=CS), 139.7 (H*C*=CCH$_2$), 134.1 (2ArC), 133.0 (2ArCH), 132.2 (2ArCH), 132.0 (2ArCH), 131.7 (2ArCH), 129.8 (ArC), 128.7 (ArC), 123.3 (ArCBr), 123.1 (ArCBr), 31.7 (*C*H$_2$). Mass spectrum: HRMS (ES$^-$) found 494.8901, $C_{19}H_{13}O_4SBr_2$ calculated 494.8901.

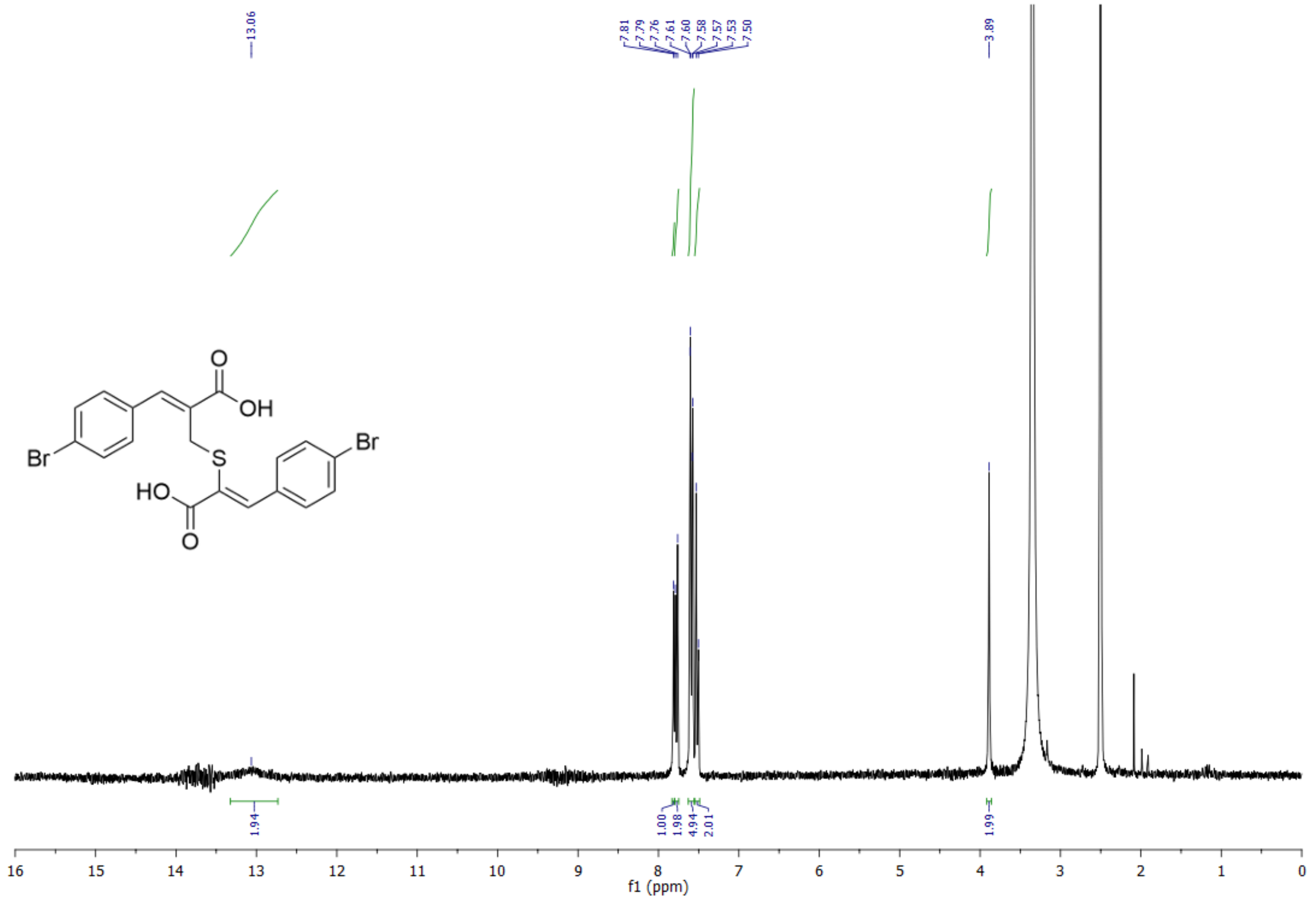

(Z)-3-(4-Bromophenyl)-2-(((Z)-2-(3-chlorophenyl)-1-carboxyallyl)thio)acrylic acid

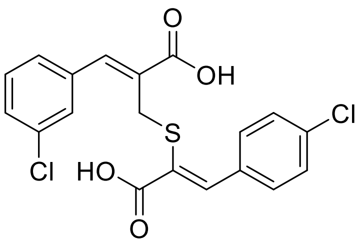

Yield: 47 %. m. p. 234 – 237 °C. δ_H (400 MHz, DMSO): 13.06 (2 H, s, COO*H*), 7.89 – 7.82 (3 H, m, 3 ArCH), 7.63 (2 H, s, 2 ArCH), 7.53 (1 H, d, *J* = 7.3, ArCH), 7.49 – 7.39 (4 H, m, 4 ArCH), 3.90 (2 H, s, C*H*$_2$). δ_C (75 MHz, DMSO): 168.4 (*C*OOH), 166.8 (*C*OOH) 141.8 (H*C*=CCH$_2$), 139.4 (H*C*=CS), 136.9 (ArC), 134.5 (ArC), 133.9 (ArCCl), 133.7 (ArCCl), 132.8 (2 ArCH), 130.9 (ArCH), 130.5 (ArC), 129.6 (ArCH), 128.8 (2 ArCH), 128.7 (ArCH), 128.6 (ArC), 31.5 (*C*H$_2$). Mass spectrum: HRMS (ES$^-$) found 406.9899, $C_{19}H_{13}O_4SCl_2$ calculated 406.9912.

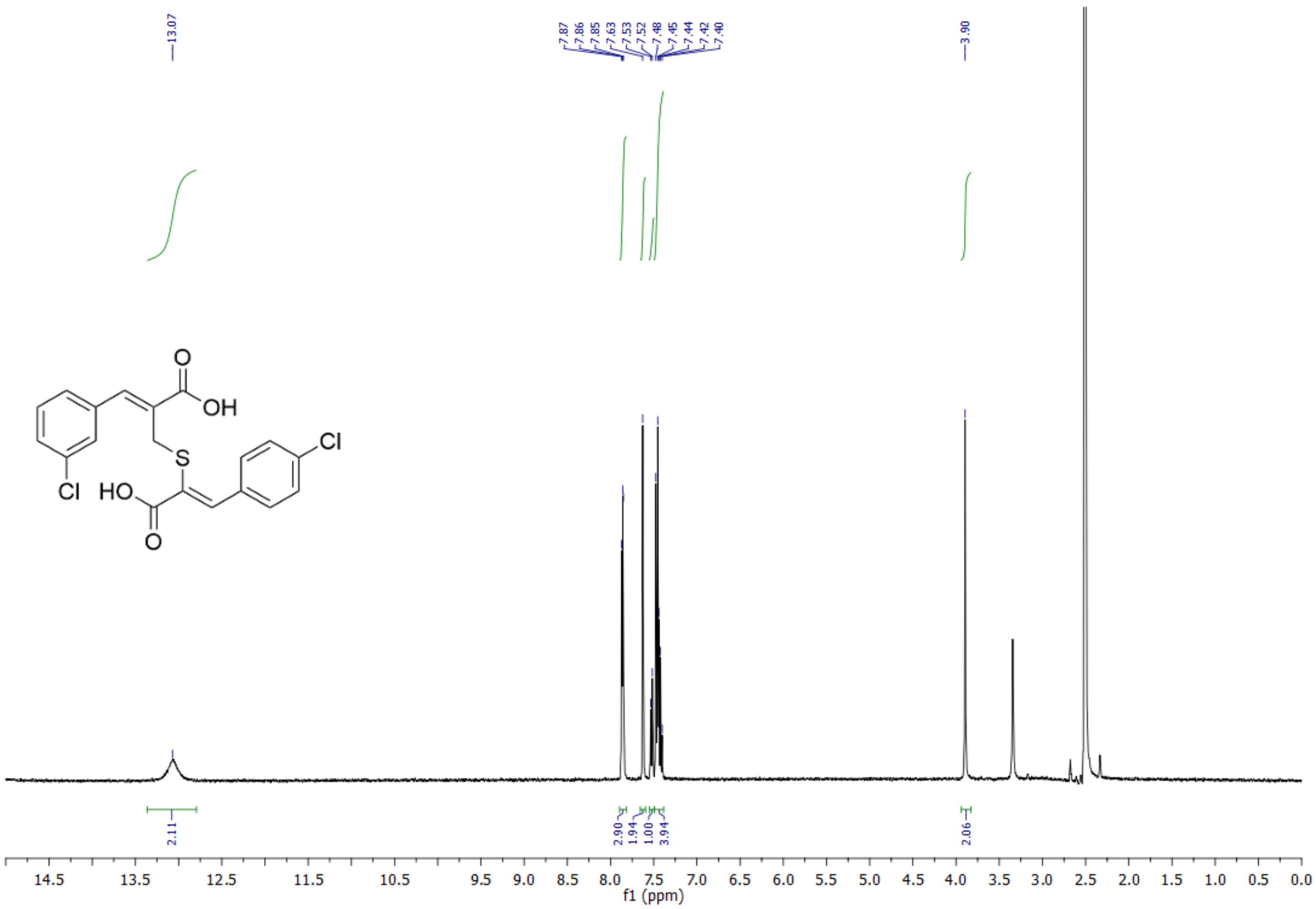

(Z)-3-(5-Bromoindol-3-yl)-2-(((Z)-3-(3-chlorophenyl)-2-carboxyallyl)thio)acrylic acid

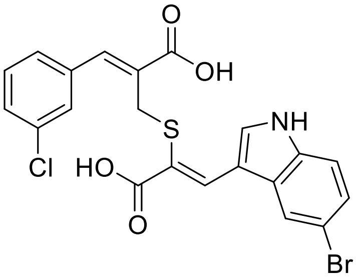

Yield: 41%. m. p 195 – 198 °C.δ_H (500 MHz, DMSO): 12.63(2 H, s, COO*H*), 12.05 (1 H, s, NH), 8.56 (1 H, d, *J* = 3.0, ArCH), 8.21 (1 H, s, C*H*=CS), 7.92 (1 H, d, *J* = 2.0, ArCH), 7.64 (1 H, s, ArCH), 7.61 (1 H, s, C*H*=CCH$_2$) 7.60 (1 H, d, *J* = 7.0, ArCH), 7.45 (1 H, d, *J* = 8.5 ArCH), 7.39 (2 H, m, 2 ArCH), 7.33 (1 H, dd, J_1 = 8.5, J_1 = 2.0, ArCH), 3.91 (2 H, s, C*H*$_2$). δ_C (125 MHz, DMSO): 167.8 (*C*OOH), 167.0 (*C*OOH), 139.0 (H*C*=CCH$_2$), 136.5 (H*C*=CS), 134.3 (ArC), 133.4 (ArCCl) 131.4 (ArCH), 131.3 (ArC), 130.7 (ArCH), 130.0 (ArC) 129.8 (ArCH), 129.2 (ArCH), 128.7 (ArCH), 128.3 (ArC), 125.5 (ArCH), 120.8 (ArCH), 118.9 (ArCBr), 114.6 (ArCH), 113.3 (ArC), 110.1 (ArC), 30.8 (CH$_2$). Mass spectrum: HRMS (ES$^-$) found 489.9521, $C_{21}H_{14}NO_4SClBr$ calculated 489.9515.

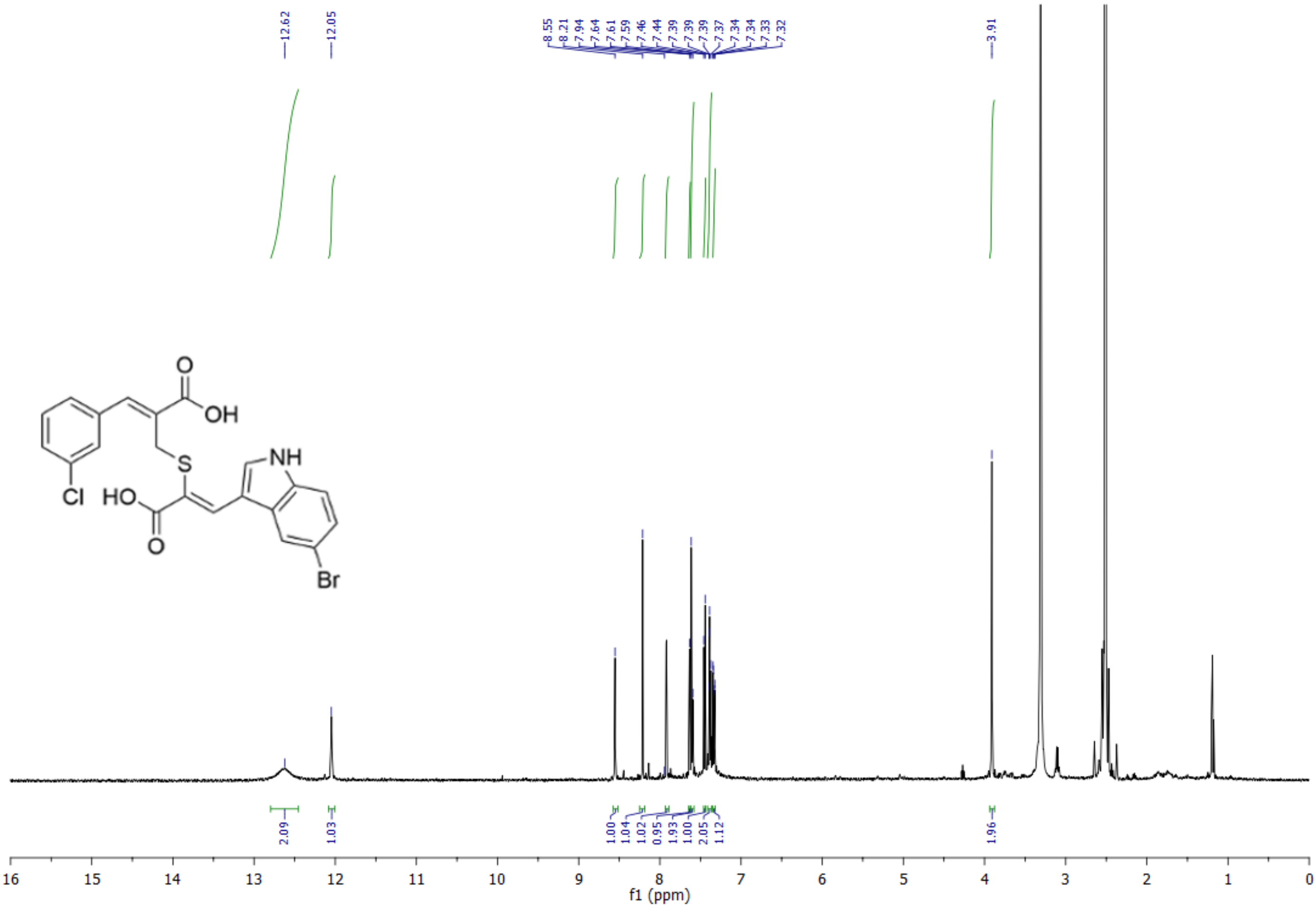

Inhibition of calpain-I with (2Z,2'Z)-2,2'-disulfanediylbis(3-(6-bromoindol-3-yl)acrylic acid) (4).

(2*Z*,2'*Z*)-2,2'-disulfanediylbis(3-(6-bromoindol-3-yl)acrylic acid) (4) was tested against calpain-I with the FRET-based inhibition assay along with (*Z*)-3-(6-bromoindol-3-yl)-2-mercaptoacrylic acid that oxidised *in situ* to form the disulfide during the assay.

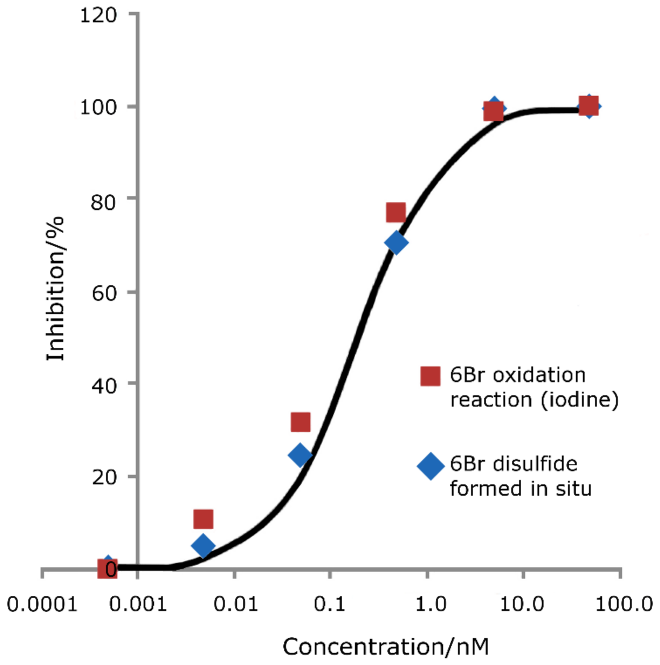

Figure S8: Dose response curve for inhibition of calpain-I by (2*Z*,2'*Z*)-2,2'-disulfanediylbis(3-(6-bromoindol-3-yl)acrylic acid) (**4**) prepared from the iodine mediated oxidation reaction and *in situ* prior to the assay (IC_{50} = 0.2 μM).

Thioether-PEF(S) X-ray crystallography data

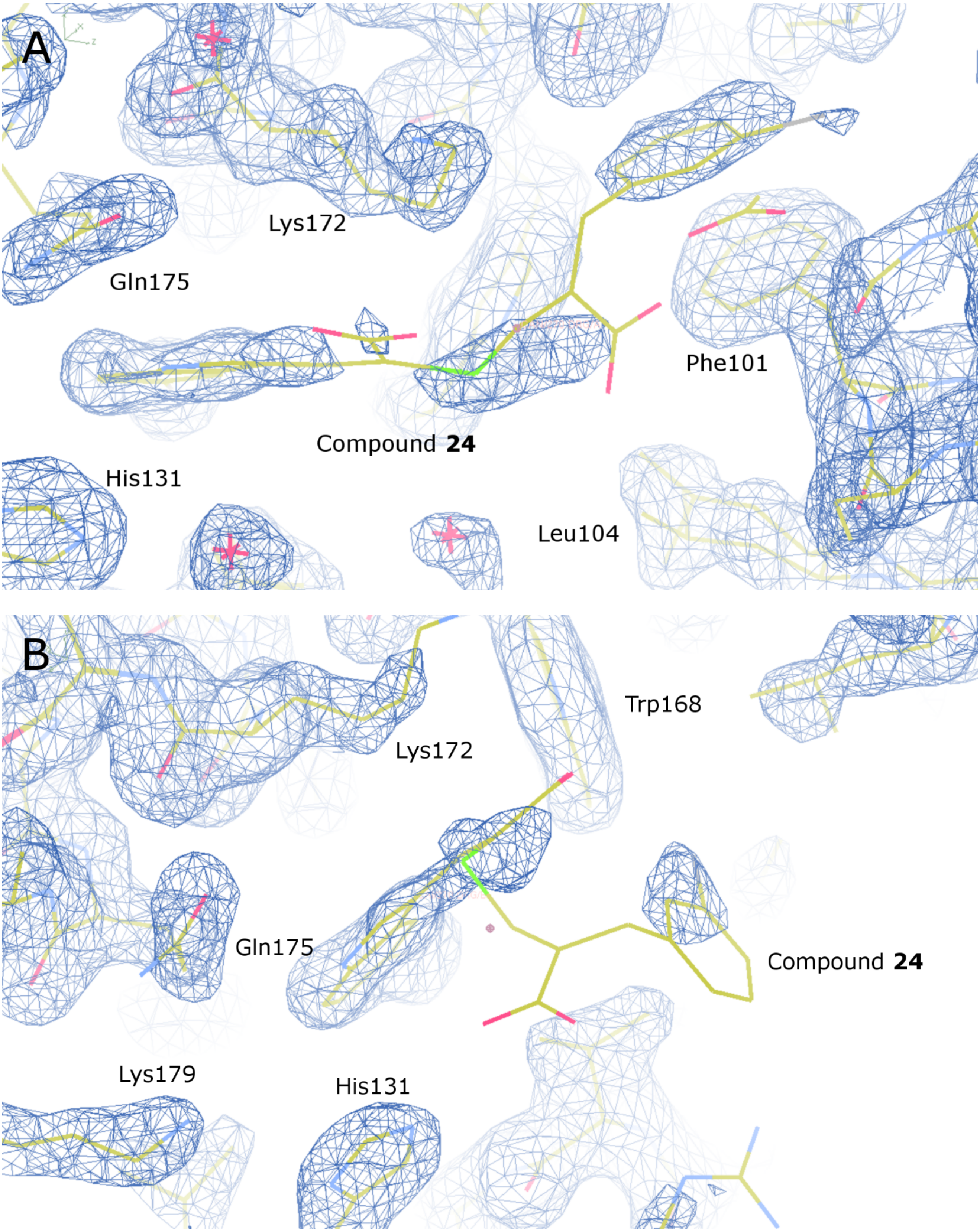

Figure S9: A stick representation of 3-(Z)-3-(5-Bromoindol-3-yl)-2-(((Z)-3-(3-chlorophenyl)-2-carboxyallyl)thio)acrylic acid (**24**) bound to PEF(S) with the $2F_0$-F_c map represented in blue and contoured to 1.0σ. In chain A (A) the occupancy of the density by **24** is 34% and chain B (B) occupancy of the density by **24** is 25%. The resolution of the structure is 1.49 Å.

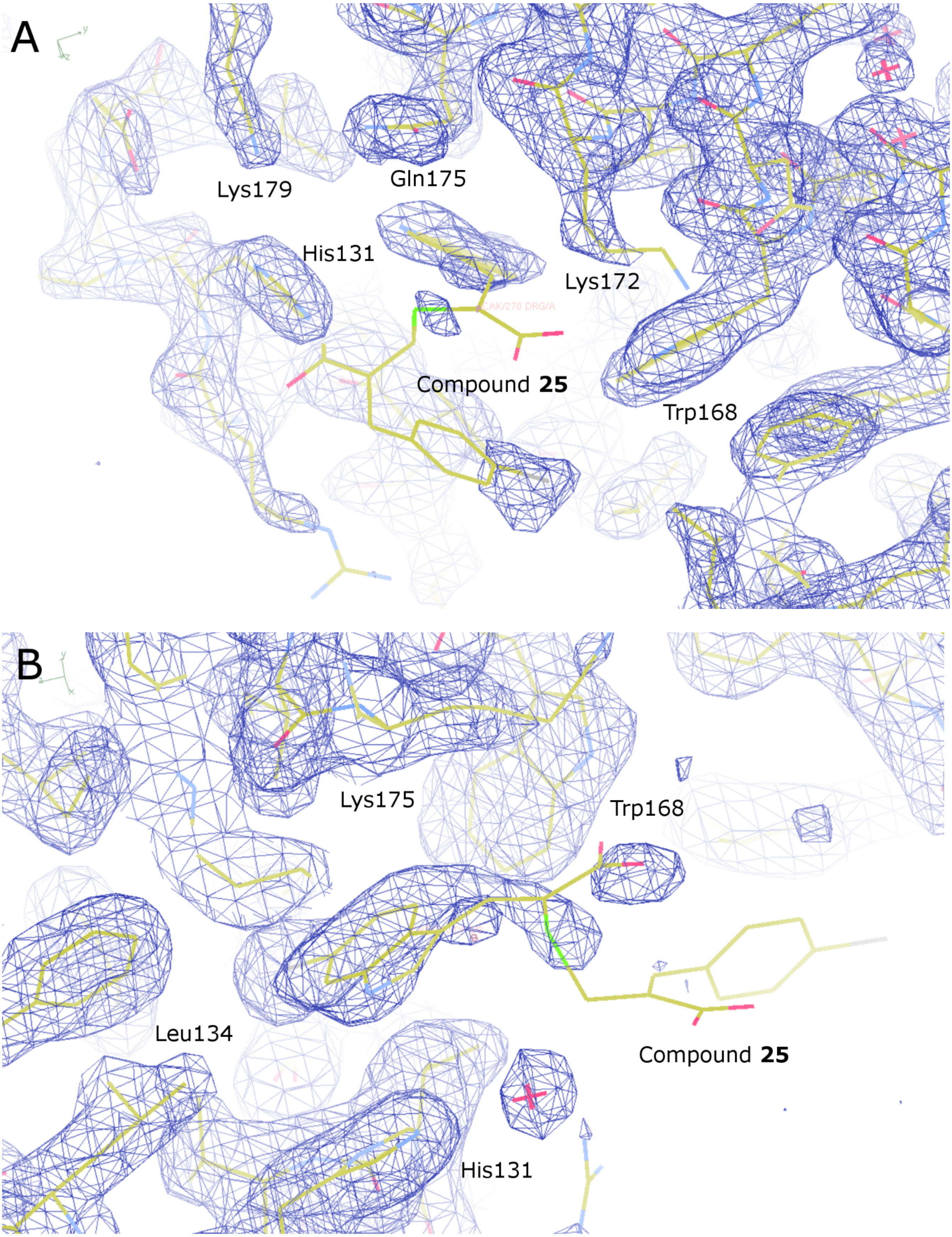

Figure S10: A stick representation of (Z)-3-(6-Bromoindol-3-yl)-2-(((Z)-3-(4-bromophenyl)-2-carboxyallyl)thio)acrylic acid (**25**) bound to PEF(S) with the $2F_0$-F_c map represented in blue and contoured to 1.0σ. In chain A (A) the occupancy of the density by **25** is 17% and chain B (B) occupancy of the density by **25** is 24%. The resolution of the data is 1.92 Å.

Oxidised PD150606 (23) crystal complex PEF(S)

The data statistics for the structure of PEF(S) - **23**.

	**PEF(S)-23**
**Data Collection**	
X-ray source	DLS I03
Space Group	$P12_11$
Cell Dimensions	
a, b, c (Å)	50.03, 79.65, 57.16
α, β, γ (°)	90.00, 91.81, 90.00
Wilson B-factor (A^2)	34.9
Resolution (Å)	34.47-1.97 (1.68-1.64)
Unique Reflections	31256 (2325)
Multiplicity	3.6 (3.8)
Completeness (%)	98.4 (99.0)
Mean $I/\sigma I$	9.5 (1.4)
R_{merge}	0.058 (0.729)
**Refinement**	
Resolution/Å	34.47-1.97
No. Reflections	29754
R_{work} / R_{free}	0.199/0.234
No. atoms	
Protein	2825
Ligand/ion	117
Water	115
B-factors/$Å^2$	
Protein	49.4
Ligands	73.9
Ions	47.7
Water	50.2
r.m.s.deviations	
Bond length/Å	0.019
Bond angles/°	1.963
PDB code	5D69

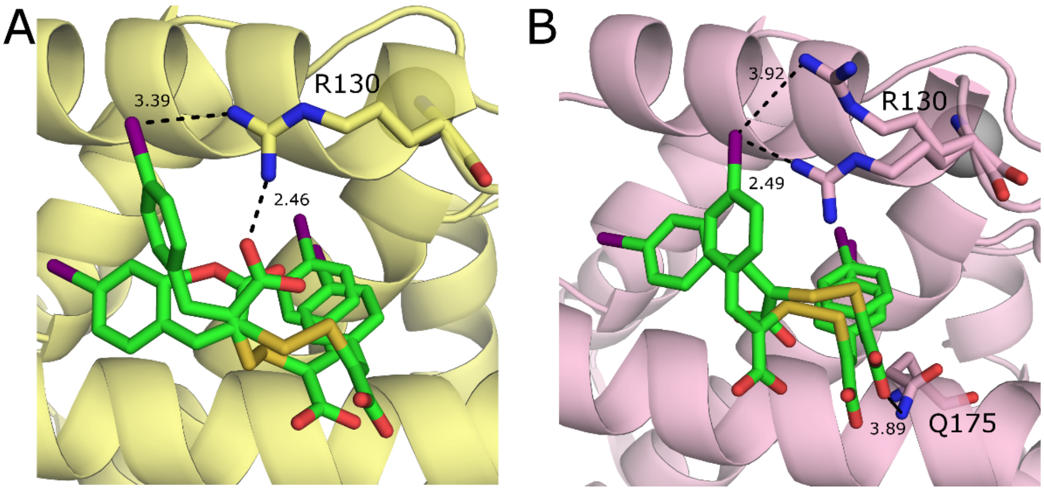

Figure S11: The hydrophilic interactions and the halogen bonds between compound **23** and PEF(S). Chain A of the homodimeric PEF(S) is represented in yellow (A) and chain B is represented in pink (B). Distances shown are in Angstroms.

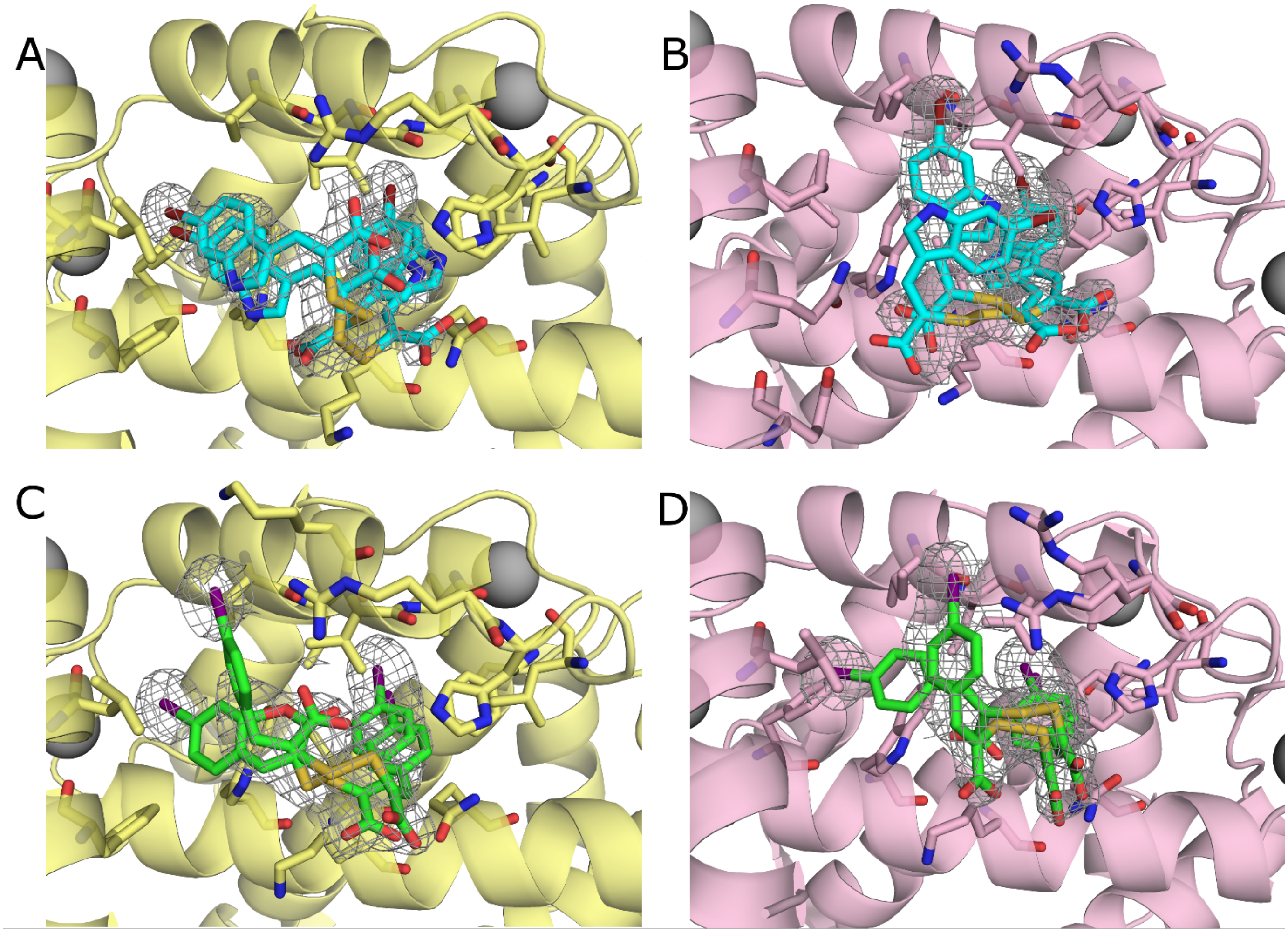

Figure S12: A comparison of the electron density observed for the ligands **4** (cyan) and **23** (green) bound to chain A (yellow, A and C) and chain B (pink, B and D) of the homodimer PEF(S). The electron density map is contoured to 1.0σ for **4** and 0.5σ for **23**. The protein is shown in cartoon form, residues within 4.0 Å of the ligand and the ligands are represented in stick form.

References

1. G. D. Lin, D. Chattopadhyay, M. Maki, E. Takano, M. Hatanaka, L. DeLucas and S. V. L. Narayana, *Acta Crystallogr. Sect. D-Biol. Crystallogr.*, 1997, 53, 474-476.
2. G. Winter, *J. Appl. Crystallogr.*, 2010, 43, 186-190.
3. P. Evans, *Acta Crystallogr. Sect. D-Biol. Crystallogr.*, 2006, 62, 72-82.
4. P. R. Evans and G. N. Murshudov, *Acta Crystallogr. Sect. D-Biol. Crystallogr.*, 2013, 69, 1204-1214.
5. E. Potterton, P. Briggs, M. Turkenburg and E. Dodson, *Acta Crystallogr. Sect. D-Biol. Crystallogr.*, 2003, 59, 1131-1137.
6. A. J. McCoy, *Acta Crystallogr. Sect. D-Biol. Crystallogr.*, 2007, 63, 32-41.
7. S. E. Adams, P. J. Rizkallah, D. J. Miller, E. J. Robinson, M. B. Hallett and R. K. Allemann, *J. Struct. Biol.*, 2014, 187, 236-241.
8. P. Emsley and K. Cowtan, *Acta Crystallogr. Sect. D-Biol. Crystallogr.*, 2004, 60, 2126-2132.
9. G. N. Murshudov, A. A. Vagin and E. J. Dodson, *Acta Crystallogr. Sect. D-Biol. Crystallogr.*, 1997, 53, 240-255.
10. A. W. Schuttelkopf and D. M. F. van Aalten, *Acta Crystallogr. Sect. D-Biol. Crystallogr.*, 2004, 60, 1355-1363.
11. S. Mittoo, L. E. Sundstrom and M. Bradley, *Anal. Biochem.*, 2003, 319, 234-238.
12. S. Bailey, *Acta Crystallogr. Sect. D-Biol. Crystallogr.*, 1994, 50, 760-763.
13. S. E. Adams, C. Parr, D. J. Miller, R. K. Allemann and M. B. Hallett, *MedChemComm*, 2012, 3, 566-570.

14. M. Kirihara, Y. Asai, S. Ogawa, T. Noguchi, A. Hatano and Y. Hirai, *Synthesis-Stuttgart*, 2007, DOI: 10.1055/s-2007-990800, 3286-3289.
15. Y. Zulykama and P. T. Perumal, *Tetrahedron Lett.*, 2009, 50, 3892-3896.
